# Modified Shen-Yan-Fang-Shuai formula attenuates diabetic kidney disease progression via regulation of HIF-1α-mediated mitochondrial energy metabolism

**DOI:** 10.1186/s13020-025-01298-5

**Published:** 2026-01-09

**Authors:** Bingnan Di, Yaotan Li, Jinyan Wei, Yizhen Han, Jinyi Hou, Xinghua Zhang, Xiaochang Wu, Weijing Liu, Huijuan Zheng, Yaoxian Wang

**Affiliations:** 1 Dongzhimen Hospital Affiliated to Beijing University of Chinese Medicine, Beijing, 100700 China; 2 Hangzhou Traditional Chinese Medicine Hospital, Wulin Campus, Hangzhou, 310013 China; 3 Renal Research Institution of Beijing University of Chinese Medicine, Beijing, 100029 China; 4 Henan University of Chinese Medicine, Zhengzhou, 450046 China; 5 Shaanxi Provincial People’s Hospital, Shanxi, 710005 China; 6 Beijing Anorectal Hospital, Beijing, 100032 China

**Keywords:** Diabetic kidney disease, HIF-1α, Metabolic reprogramming, Mitochondrial dysfunction, Traditional Chinese medicine, Glycolysis, Oxidative phosphorylation

## Abstract

**Background:**

Diabetic kidney disease (DKD) represents a major global health burden, affecting 20–40% of diabetic patients worldwide. Metabolic reprogramming mediated by hypoxia-inducible factor-1α (HIF-1α) plays a central role in DKD pathogenesis, yet effective therapeutic strategies remain limited. The Modified Shen-Yan-Fang-Shuai formula (M-SYFSF), a traditional Chinese medicine formulation, has demonstrated clinical efficacy in DKD treatment, but its underlying mechanisms remain unclear.

**Methods:**

A DKD model was established using streptozotocin-induced diabetic rats following unilateral nephrectomy. Thirty rats were randomly divided into sham operation, model, and M-SYFSF treatment groups (n = 10/group). M-SYFSF was administered at 11.34 g/kg/d for 12 weeks. Renal function, histopathology, oxidative stress markers, and metabolic parameters were assessed. Human proximal tubular epithelial cells (HK-2) were treated with advanced glycation end products under hypoxic conditions to establish an in vitro DKD model. HIF-1α overexpression and knockdown experiments were performed to investigate molecular mechanisms. Key glycolytic enzymes, mitochondrial dynamics proteins, and bioenergetic parameters were analyzed using Western blot, immunohistochemistry, immunofluorescence, and metabolic assays.

**Results:**

M-SYFSF treatment significantly improved renal function parameters, reducing serum creatinine (*p* < 0.001) and proteinuria (*p* < 0.001) while ameliorating characteristic DKD histopathological changes. M-SYFSF effectively suppressed HIF-1α expression and nuclear translocation, accompanied by consistent downregulation of key glycolytic enzymes including hexokinase 2, lactate dehydrogenase, and pyruvate dehydrogenase kinase 1. Metabolic analysis revealed that M-SYFSF promoted a shift from glycolysis toward oxidative phosphorylation, restoring mitochondrial ATP production capacity. Transmission electron microscopy demonstrated that M-SYFSF preserved mitochondrial ultrastructure and improved mitochondrial respiratory chain complex activities (I, III, and IV; all *p* < 0.01). M-SYFSF treatment enhanced mitochondrial fusion by upregulating Mfn1 and Mfn2 while suppressing fission proteins Drp1 and Fis1. HIF-1α overexpression experiments confirmed that M-SYFSF’s metabolic and mitochondrial protective effects were mediated through HIF-1α pathway modulation. Additionally, M-SYFSF significantly reduced oxidative stress markers, including 8-OHdG and malondialdehyde levels (*p* < 0.001), while enhancing antioxidant enzyme activities.

**Conclusions:**

M-SYFSF exerts significant nephroprotective effects in diabetic kidney disease by targeting HIF-1α-mediated metabolic reprogramming. The therapeutic mechanisms involve suppression of pathological glycolytic metabolism, restoration of mitochondrial function and dynamics, and enhancement of antioxidant capacity. These findings provide mechanistic validation for M-SYFSF as a promising multi-target therapeutic approach for diabetic kidney disease management and establish HIF-1α as a key therapeutic target for metabolic intervention in DKD treatment.

**Supplementary Information:**

The online version contains supplementary material available at 10.1186/s13020-025-01298-5.

## Introduction

Diabetic kidney disease (DKD) has become a catastrophic global health issue and is now recognized as the primary cause of chronic kidney disease (CKD), end-stage renal disease (ESRD) worldwide [1, 2]. An estimated 537 million people around the world had diabetes in 2021, a figure projected to have risen to783 million by 2045 [3]. DKD affects 20–40% of all diabetics and the worldwide burden of DKD has increasedsteadily between 1990–2021, with a further growth predicted up to 2050 [4, 5], posing an unprecedented healthcare challenge requiring revolutionary therapeutic interventions.

The pathogenesis of DKD is characterized by intricate metabolic, hemodynamic and inflammatory pathways leading to progressive renal disease. Critical to the disease process is metabolic reprogramming bywhich renal cells undergo a significant transition from oxidative phosphorylation to glycolysis, in adaptation under diabetic milieu [6, 7]. These changes accumulate in part because mitochondrial dysfunction induces pathway adjustments within kidneycells that further promote progression of DKD and creates a proinflammatory environment that then results in the recruitment of immune cells to renal tissue [8]. This has created an awareness that DKD is not predominantly a state following hyperglycemia but complex disease with major metabolic and immune underpinnings [9, 10].

Hypoxia-inducible factor-1 (HIF-1) is now recognized as a major transcriptional mediator in the pathogenesis of DKD and functions as a “molecular switch”, regulating cellular responses to hypoxic and metabolic stress. In the DKD, HIF signaling has cell-specific outcomes; increased glomerular HIF activities drive glomerulosclerosis and albuminuria, allwhile tubular HIF induction preserves mitochondria performance and attenuates diabetes-induced tissue hypoxia and fibrosis [11]. The current evidence indicates that HIF-1 is atherapeutic target for SGLT2 inhibitors, which protect against diabetic kidney via their role in repression of hypoxia-induced HIF-1 accumulation by inhibition of mitochondrial oxygen consumption [12]. HIF-1 can directly regulate the expression of the glycosylating enzymes such as HK2, LDH and PDK1 which then help to switch metabolism towards glycolysis [13, 14].

Mitochondrial impairment is one of the fundamentalmechanisms involved in the progress of DKD. As the kidney isan organ with high-energy needs, it is very susceptible to mitochondrial damage in the form of defects in oxidative phosphorylation, ROS overproduction, and disturbances in protein content of mitochondria [15, 16]. It has been demonstrated that mitochondrial DNA mutations, reduced expression ofthe electron transport chain complex, impaired fusion-fission events, and dysregulation of mitophagy have all been summarized in DKD [17]. Those changes establish a vicious self-catalyzing cycle in which defective mitochondria leads to oxidative stress, leading to even more damaged mitochondria and sustaining the cell injury [18, 19].

Traditional Chinese Medicine (TCM) has been shown to have unique advantagesin the treatment of DKD, and it can provide a multitarget strategy for the comprehensive management of complicated pathophysiological processes. As a complementary therapy method, Chinese herbal medicine has demonstrated effects in enhancing clinical symptoms alleviation, proteinuria reduction, renal function protection and the slow down of the progression of DKD [20, 21]. TCM decoction is a usual adjuvant treatment for DN, which has its clinical benefit and safety [22]. Mechanistic investigations have shown that pathways regulating glucose/lipid metabolism, antioxidative stress and anti-inflammation, reversal of fibrosis [23, 24], and protection of podocytes are important mechanisms for TCM in the treatment of DN as compared with conventional single target therapy [25]. The Modified Shen-Yan-Fang-Shuai formula (M-SYFSF) recipe is a TCM-based approach targeting metabolic disorder in the development of DKD. Nevertheless, the exact actionof M-SYFSF on the HIF-1 dependent metabolic reprogramming has not been fully elucidated. Considering the recent identified dominant role for HIF-1 in metabolic reprogramming of DKD and the potential multitarget therapeutic property of TCM, exploration on the crosstalk between M-SYFSF and HIF-1 signaling may become an important research direction. Thus, theobjective of our present study is to systematically explore the renal protective effects of marein SYFSF on DKD and for the first time to focus on revealing whether HIF-1 mediated metabolic reprogramming contributes to its therapeutic potential.

## Materials and methods

### Network pharmacology analysis

In order to uncover the underlying action mode of M-SYFSF in treating DKD, a systemic network pharmacology approach was used. The active ingredients of M-SYFSF were mainly collected from the TCM Systems Pharmacology Database and Analysis Platform (TCMSP,https://old.tcmsp-e.com/tcmsp.php). For the compounds not found in TCMSP, further databases such as PubChem(https://pubchem.ncbi.nlm.nih.gov/), ChEMBL web (https://www.ebi.ac.uk/chembl/) and literatures were consulted for a complete coverage. The databases were filtered using the criterion of oral bioavailability (OB) ≥ 30% anddrug-likeness (DL) ≥ 0.18 in order to guarantee pharmacological relevance. Meanwhile, the primarybioactive constituents in M-SYFSF extract were confirmed by UPLC-ESI–MS/MS according to our previous study [26]. The Swiss Target prediction (http://www.swisstargetprediction.ch/) and SuperPred (https://prediction.charite.de/) databases were used to predict target proteins of the selected active compounds, only targets with confidence scores greater than 0.5 were considered. Disease-related targets for DKD were systematically collected from GeneCards (https://www.genecards.org/), Online Mendelian Inheritance in Man (OMIM, https://omim.org/), and National Center for Biotechnology Information (NCBI, https://www.ncbi.nlm.nih.gov/) databases using search terms “diabetic kidney disease”. For analysis, we chose highly relevant targets with scores > 10 in GeneCards. Venn diagram analysis was used to explore common targets of the activen compounds in M-SYFSF and DKD as potential therapeutic target. Pathway enrichment analysis of Kyoto Encyclopedia of Genes and Genomes (KEGG) was carried out by Database for Annotation, Visualization and Integrated Discovery (DAVID; https://david.ncifcrf.gov/). Pathways with *P* < 0.05 and false discovery rate (FDR) < 0.05 were considered statistically significant. The bubble plots were applied to visually demonstratethe correlation between pathway significance and proportion of enriched genes.

### Molecular docking analysis

To explore the binding mode of major active compounds of M-SYFSF with target proteins screened out by network pharmacology analysis, molecular docking were used by AutoDock Vina (version 1.1.2). The 3D structures of the target proteins containing HIF-1 (PDB ID: 4H6J), DRP1 (PDB ID: 4BEJ), and MFN1 (PDB ID: 3ZUW) were downloaded from Protein Data Bank (https://www.rcsb.org/) and preprocessed through AutoDockTools. Chemical structures of active compounds including quercetin, kaempferol, and formononetin were obtained from the PubChem database (https://pubchem.ncbi.nlm.nih.gov/) and optimized using ChemDraw software. The molecular docking simulations (exhaustiveness = 8, number of runs = 10, energy range = 3 kcal/mol) were implemented. Docking was analyzed by binding affinity(kcal/mol) and molecular interactions were depicted by PyMOL software. Compounds with binding affinity scores ≤ −5.0 kcal/mol were considered to have good binding potential.

### Main reagents

Modified Shen-Yan-Fang-Shuai formula (M-SYFSF) was designed under the guidance of traditional Chinese medicine theory and produced by standard extraction methods. Table 1 shows the herbal composition and dosage ratio used in M-SYFSF. All botanical names have been checked against the World Flora Online database (www. worldfloraonline. org) and authenticated following the Chinese Pharmacopoeia (https://ydz.chp.org.cn) in order to guarantee taxonomic authenticity even pharmacological standardization. Streptozotocin (STZ) was purchased from Sigma-Aldrich (St. Louis, MO, USA). H&E staining kit (G1076), PAS staining kit (G1008), and Masson’s trichrome staining kit (G1006) were purchased from Solarbio Science & Technology Co., Ltd. (Beijing, China). FastPure^®^ Cell/Tissue Total RNA Isolation Kit (RC101), HiScript^®^ III All-in-one RT SuperMix Perfect for qPCR (R333), and Taq Pro Universal SYBR qPCR Master Mix were purchased from Vazyme Biotech (Jiangsu, China). Cell Counting Kit-8 (CCK-8, GK10001) was purchased from GlpBio Technology Co., Ltd. (Shanghai, China). GSH assay kit (A006-2-1), MDA assay kit (A003-1–2), CAT assay kit (A007-1-1), and ATP assay kit (A095-1-1) were purchased from Nanjing Jiancheng Bioengineering Institute (Jiangsu, China). Mitochondrial respiratory chain complex I, III, and IV activity assay kits were purchased from Beyotime Biotechnology (Shanghai, China). Oligomycin and 2-deoxy-D-glucose (2-DG) were purchased from Sigma-Aldrich (St. Louis, MO, USA).

**Table 1:** Herb composition in Modified Shen-Yan-Fang-Shuai Formula

Number	Chinese name	English name	Latin name	Medicinal Parts	Voucher number	Weight (g)
1	Huang Qi	Astragalus root	*Astragalus mongholicus* Bunge	Root	ShanDong 717010401	30
2	Dang Gui	Chinese angelica	*Angelica sinensis* (Oliv.) Diels	Root	GanSu 717030103	10
3	Hai Zao	Seaweed	*Sargassum pallidum* (Turn.) C. Ag	Dried algae	ZheJiang 7130121	10
4	Bie Jia	Turtle shell	*Trionyx sinensis* Wiegmann	Carapace	HuBei 717041001	10
5	Mu Li	Oyster shell	*Ostrea gigas* Thunberg	Shell	ShanDong 70115010101	15
6	San Qi	Notoginseng	*Panax notoginseng* (Burk.) F.H. Chen	Root	YunNan 71109	3
7	Shu Di Huang	Prepared rehmannia	*Rehmannia glutinosa* (Gaertn.) DC	Root	HeNan 717030401	30

### Preparation of M-SYFSF extract

All herbal materials used in M-SYFSF were purchased from the Department of Pharmacy, Dongzhimen Hospital, Beijing University of Chinese Medicine (Beijing, China) and authenticated by pharmacognosy experts from the Department of Pharmacy, Beijing University of Chinese Medicine, according to the Chinese Pharmacopoeia (2020 Edition). Voucher specimens are available upon request.

M-SYFSF was prepared in accordance with principles oftraditional Chinese medicine from our previous optimization studies. We further indicated the optimal therapeutic dosage of M-SYFSF against DKD (11.34 g/kg/d) by an effective and systematic dose-response studies through network pharmacology, combinedwith animal experiments [26], which laid a solid foundation for the following mechanism-based study. The herbal combination except for Panax notoginseng (105 g crude drug in total: 30 g *Astragalus mongholicus*, 10 g *Angelica sinensis*, 10 g *Sargassum pallidum*, 10 g *Trionyx sinensis*, 15 g *Ostrea gigas* and 30 g *Rehmannia glutinosa*) was steeped in distilled water (1:10 w/v) at room temperature for 30 min. The Decoction The mixed upper decoction was boiling underreflux for 30 min, and the first decoction was obtained by filtrating it. The residue was refilled with 840 ml of distilled water (8 times the volume), boiled, and decocted for another 30 min toobtain the second decoction. The two decoctions were mixed together and filtered through 4 layers of sterile gauze to remove coarse material, then passed sequentially through a 200-mesh stainlesssteal screen (75 m in particle size) so that the solution was clear and uniform. This filtered extract was concentrated under vacuum at 60  C to about 105 ml using a rotary evaporator. After cooling down to room temperature, 3 g of Panax notoginseng powder (passed through an 80-mesh sieve) was mixed with the concentrated extractand stirred for 10 min to dissolve it fully and evenly distribute it into the solution. The final extracted concentration was adjusted to 1 g crude drug equivalent per milliliter (1 g/ml, indicating 108 g of the total crude drugs in 108 mlfinal volume). The concentrated extract was filtered through single-layer sterile gauze and aliquoted into sterile 50 ml centrifuge tubes, then stored at −20 °C. For each dosing, the required volume was withdrawn from a tube using sterile technique, thawed at 4 °C for 1 h, and warmed to room temperature before administration. The tube was immediately returned to −20 °C after use. Each 50 ml tube was used for approximately one week of dosing to limit the number of freeze-thaw cycles.

### Animal experiments

Thirty-five SPF-grade male Sprague–Dawley rats, aged 8–10 weeks and weighing 200 ± 20 g, were purchased from Beijing Vital River Laboratory Animal Technology Co., Ltd. (Beijing, China). All animals were housed under controlled conditions (24 ± 2 °C, 12 h light/dark cycle) with free access to standard chow and water. After one week of acclimatization, rats were randomly divided into two groups: sham operation group (n = 10) and diabetes modeling group (n = 25). This study was approved by the Ethics Committee for Experimental Animals of Dongzhimen Hospital, Beijing University of Chinese Medicine (Approval No. DZMYY24-19).

### Establishment of diabetic kidney disease model

The diabetes modeling group (n = 25) underwent left unilateral nephrectomy under anesthesia (2% pentobarbital sodium, 40 mg/kg, intraperitoneally). Briefly, after a left flank incision, the left kidney was exposed, the renal pedicle was ligated, and the kidney was excised. The surgical site was closed in layers. Animals were monitored twice daily for 7 days to assess body weight, food and water intake, locomotor activity, and pain-related behaviors. Comprehensive postoperative care was provided, including warm bedding, moistened food, and subcutaneous fluids as needed to support recovery. All animals resumed normal activity within 24–48 h, and no signs of severe pain or distress were observed. The sham operation group (n = 10) underwent the same surgical procedure including anesthesia, flank incision, and exposure of the left kidney without excision, followed by layer-by-layer closure, with the same postoperative analgesia protocol. One week after surgery, rats in the diabetes modeling group received a single intraperitoneal injection of streptozotocin (STZ, 50 mg/kg) dissolved in freshly prepared 0.1 M citrate buffer (pH 4.5). The sham operation group received an equivalent volume of citrate buffer. Blood glucose levels were monitored every 3 days using a glucometer. Rats with fasting blood glucose levels ≥ 16.7 mmol/L for three consecutive measurements were considered successfully diabetic.

To confirm early kidney damage, 24-h urine samples were collected on day 14 post-STZ injection, and urinary albumin levels were measured. Rats with both sustained hyperglycemia (≥ 16.7 mmol/L) and elevated urinary albumin (≥ 30 mg/24 h) were considered as successful DKD models. Among the 25 rats in the diabetes modeling group, 20 rats successfully developed DKD (modeling success rate: 80%). Five rats that did not meet the criteria (3 rat died during surgery, 2 rats failed to develop sustained hyperglycemia) were excluded from the study.

### Drug administration and experimental grouping

The successfully modeled diabetic rats (n = 20) were randomly divided into two groups: model group (n = 10) and M-SYFSF treatment group (n = 10). The sham operation group (n = 10) remained unchanged. Therefore, the final experimental design consisted of three groups with 10 rats each: sham operation group, model group, and M-SYFSF group. Drug administration began immediately after randomization and continued for 12 weeks. The M-SYFSF group received M-SYFSF at a dose of 11.34 g/kg/d, administered by oral gavage twice daily. The sham operation and model groups received equivalent volumes of normal saline twice daily by oral gavage. Body weight was monitored weekly, and drug dosages were adjusted accordingly. The complete experimental design, including initial allocation, modeling outcomes, randomization, and final sample sizes, is summarized in Supplementary Figure S1.

### Serum and urine biochemical parameter analysis

After 12 weeks of intervention, rats were fasted for 12 h with free access to water before specimen collection. Body weight and kidney weight were measured and recorded. Blood samples were collected from the abdominal aorta under anesthesia (45 mg/kg pentobarbital sodium intraperitoneally), centrifuged at 3500 rpm for 10 min, and serum was stored at −80 °C until analysis. 24-h urine samples were collected using metabolic cages at weeks 0, 4, 8, and 12, centrifuged at 3500 rpm for 10 min, and the supernatant was stored for subsequent analysis. Serum and urine creatinine levels were measured using commercial assay kits according to the manufacturer’s instructions. IL-6 levels were quantified using rat IL-6 ELISA kit (Bioswamp, RA20607). Urinary protein and urinary albumin were detected using commercial kits and rat microalbuminuria ELISA kits (Elabscience, E-EL-R0025), respectively. Creatinine clearance and urinary albumin/creatinine ratio (UACR) were calculated according to standard formulas for evaluating renal function.

### Histological analysis of kidney tissues

Kidney tissues were fixed in 4% paraformaldehyde, embedded in paraffin, and sectioned (4 μm) for routine histological examination. H&E staining was used to evaluate general morphology, PAS staining was employed to assess glycogen deposition, and Masson’s trichrome staining was used to evaluate collagen deposition and fibrosis. Quantitative analysis was performed using ImageJ software.

### Transmission electron microscopy

Renal cortical fragments were fixed in 2.5% glutaraldehyde and washed with PBS 3 times (15 min each). Post-fixation was performed using 1% osmium tetroxide at room temperature for 2 h, followed by PBS washes. Dehydration was carried out through graded ethanol (30%, 50%, 70%, 80%, 95%, 100%) for 20 min each, followed by propylene oxide treatment. For embedding, tissues were infiltrated with propylene oxide: 812 embedding medium at different ratios (1:1 for 2–4 h, 1:2 overnight, then pure 812 medium for 5–8 h) at 37  C, and polymerized at 60 °C for 48 h. Semi-thin Sects. (1.5 μm) were stained with methylene blue for positioning. Ultrathin Sects. (60–80 nm) were cut and mounted on copper grids, then double-stained with 2% uranyl acetate (8 min) and 2.6% lead citrate (8 min). Images were captured using transmission electron microscopy for analysis. Mitochondrial morphological parameters including length, width, and aspect ratio were quantified using ImageJ software.

### Cell culture and treatment

Human proximal tubular epithelial cells (HK-2) were provided by the Department of Nephrology, Dongzhimen Hospital, Beijing University of Chinese Medicine. Complete culture medium was prepared by adding 27.5 ml F12 medium (containing 10 mmol/L), 5 ml fetal bovine serum (FBS), 0.5 ml penicillin–streptomycin-amphotericin B solution, and 17 ml DMEM medium (without sugar) to achieve a final glucose concentration of 5.5 mmol/L. Cells were cultured at 37 °C in a humidified atmosphere with 5% CO₂. Cell passaging was performed when cells reached 80% confluence by trypsinization and reseeding at appropriate densities. For cryopreservation, cells were frozen in medium containing DMSO: FBS at a 1:9 ratio and stored at −80 °C.

Based on the research foundation of our team and peer studies, a DKD cell model was established using AGEs (200 μg/ml) stimulation at 37 °C under hypoxic conditions (1% O₂, 5% CO₂, 94% N₂) for 48 h to simulate the DKD cellular environment [27, 28]. Experimental groups included: (1) Control group: cells cultured in complete medium at 37 °C, 5% CO₂ for 48 h; (2) Model group: cells treated with AGEs (200 μg/ml) in complete medium at 37 °C under hypoxic conditions (1% O₂, 5% CO₂, 94% N₂) for 48 h; (3) M-SYFSF group: cells treated with AGEs (200 μg/ml) and M-SYFSY (100 μg/ml) in complete medium at 37 °C under hypoxic conditions (1% O₂, 5% CO₂, 94% N₂) for 48 h.

### Metabolic function analysis

To assess cellular metabolic function, lactate production and ATP content were measured using commercial kits following the manufacturer’s protocols. For mitochondrial function analysis, cells were treated with oligomycin (1 μM) to inhibit ATP synthase or 2-deoxy-D-glucose (10 mM) to inhibit glycolysis. Mitochondrial respiratory chain complex activities (I, III, and IV) were determined using specific assay kits according to the manufacturer’s instructions.

### Immunohistochemistry and immunofluorescence

Paraffin-embedded tissue sections were deparaffinized at 60 °C for 40 min, rehydrated through graded alcohols, and washed with PBS. Antigen retrieval was performed using citrate buffer (pH 6.0) or Tris–EDTA buffer (pH 9.0) at 95 °C for 25 min. After blocking with 3% hydrogen peroxide and 5% normal goat serum, sections were incubated with primary antibodies (HIF-1α 1:100, HK2 1:500, LDH 1:1000, PDK1 1:200, TGF-β 1:200, Drp1 1:200, Mfn1 1:200, Mfn2 1:200, α-SMA 1:6000) overnight at 4 °C, followed by HRP-conjugated secondary antibodies at 37 °C for 20 min. DAB staining and hematoxylin counterstaining were performed, then sections were dehydrated, cleared, and mounted. For immunofluorescence, cells were fixed with 4% paraformaldehyde, permeabilized, and incubated with primary antibodies followed by fluorescent secondary antibodies. Images were captured using light or fluorescence microscopy, and quantitative analysis was performed using ImageJ software.

### Western blot analysis

Protein samples were extracted using RIPA lysis buffer and quantified using the BCA assay. Equal amounts of protein (30 μg) were separated by SDS-PAGE and transferred to PVDF membranes. After blocking, membranes were incubated with primary antibodies overnight at 4 °C, followed by HRP-conjugated secondary antibodies. Protein bands were detected using enhanced chemiluminescence and quantified using ImageJ software.

### Lentivirus transfection

For HIF-1α overexpression and knockdown studies, HK-2 cells at 20–30% confluence were transfected with lentiviral vectors (MOI-20) according to the manufacturer’s protocol. After 12–16 h incubation at 37 °C, cells were selected with puromycin (2 μg/ml) for 48 h to achieve nearly 100% transfection efficiency. Transfection success was confirmed by Western blot analysis of HIF-1α expression levels. Experimental groups included: (1) Vector group: cells transfected with empty vector; (2) Vector + Model group (Vector + M): vector-transfected cells treated with AGEs (200 μg/ml) under hypoxic conditions; (3) Vector + Model + M-SYFSF group (Vector + M + M-S): vector-transfected cells treated with AGEs and M-SYFSF (100 μg/ml) under hypoxic conditions; (4) HIF-1α overexpression group (OE): cells transfected with HIF-1α overexpression vector; (5) OE + Model group (OE + M): HIF-1α overexpressing cells treated with AGEs under hypoxic conditions; (6) OE + Model + M-SYFSF group (OE + M + M-S): HIF-1α overexpressing cells treated with AGEs and M-SYFSF under hypoxic conditions. All treatments were performed for 48 h.

### Biochemical assays

Glutathione (GSH), malondialdehyde (MDA), catalase (CAT), and adenosine triphosphate (ATP) levels in kidney tissues and cultured cells were measured using commercial assay kits according to the manufacturer’s protocols. Oxidative stress marker 8-OHdG was measured using ELISA kits. All assays were performed in triplicate.

### Cell immunofluorescence staining

Cells were seeded on coverslips in 24-well plates and treated for 48 h. After treatment, cells were washed with PBS (3 times, 5 min each) and fixed with 4% paraformaldehyde for 20 min. Following PBS washes, cells were permeabilized with 0.1% Triton X-100 for 20 min and blocked with 3% BSA for 30 min. Primary antibodies (Mfn1 1:500, Fis1 1:500, LDH 1:200) were incubated overnight at 4 °C, followed by fluorescent secondary antibodies (CY3-labeled, 1:300) for 50 min in the dark. DAPI nuclear staining was performed for 10 min. After final PBS washes, coverslips were mounted with anti-fade medium and images were captured using fluorescence microscopy.

### Statistical analysis

Statistical analyses and data visualization were conducted using Excel, GraphPad Prism 10, and Adobe Illustrator 2023 software. Data are expressed as mean ± standard error of the mean (SEM). The Shapiro–Wilk test was first used to assess whether data followed a normal distribution, and the Brown-Forsythe test was used to analyze homogeneity of variance. When data met the criteria for normal distribution and homogeneity of variance, one-way analysis of variance (One-Way ANOVA) was employed for comparisons between groups. If data did not meet the criteria for normal distribution or homogeneity of variance, the non-parametric Kruskal–Wallis rank sum test was used for analysis. *P* < 0.05 was considered statistically significant.

## Results

### Network pharmacology and molecular docking reveal multi-target therapeutic mechanisms of M-SYFSF

**Fig. 1:**
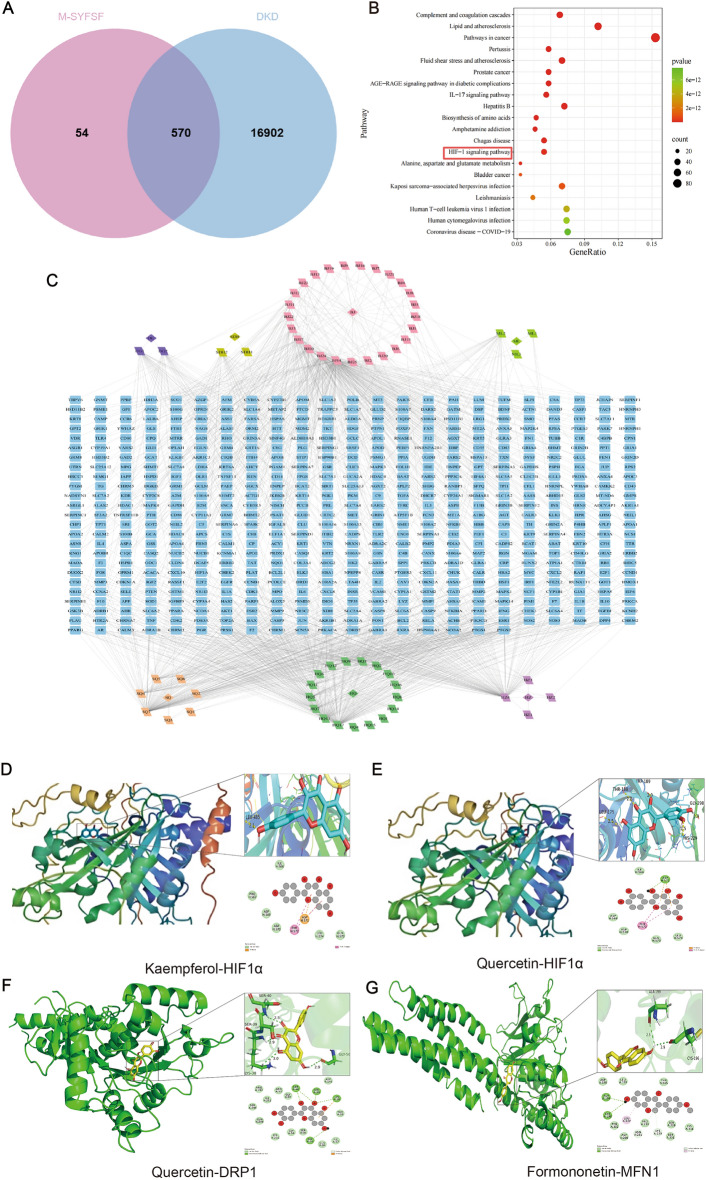
Network pharmacology and molecular docking reveal multi-target therapeutic mechanisms of M-SYFSF. **A** Venn diagram showing the overlap between M-SYFSF compounds and DKD-related targets. **B** KEGG pathway enrichment analysis bubble plot showing significantly enriched pathways. **C** Compound-target network diagram displaying the complex interactions between M-SYFSF active compounds (colored nodes) and their target proteins (blue nodes). **D** Molecular docking result of quercetin binding to DRP1 protein. **E** Molecular docking result of formononetin binding to MFN1 protein. **F**, **G** Reserved spaces for kaempferol-HIF1α and quercetin-HIF1α molecular docking results

We performed integrated network pharmacology and molecular docking analyses to investigate M-SYFSF’s molecular mechanisms in DKD. After screening based on OB ≥ 30% and DL ≥ 0.18 criteria, 624 active compounds were identified from M-SYFSF with 17,472 potential targets. For DKD, 570 disease-related targets were collected from databases using relevance score > 10. Venn diagram analysis identified 570 common targets between M-SYFSF and DKD (Fig. 1A), demonstrating substantial therapeutic overlap and multi-target potential rather than single-target mechanisms. KEGG pathway enrichment analysis revealed significant enrichment in pathways related to DKD pathogenesis (Fig. 1B). The HIF-1 signaling pathway was prominently enriched (highlighted in red box), providing bioinformatic evidence supporting our experimental findings on HIF-1α-mediated mitochondrial energy metabolism. Other relevant pathways included complement and coagulation cascades, AGE-RAGE signaling, and biosynthesis of amino acids, indicating M-SYFSF regulates DKD through modulation of metabolic reprogramming, inflammatory responses, and mitochondrial dysfunction. The compound-target network revealed complex multi-component interactions (Fig. 1C), with distinct compound clusters targeting interconnected biological pathways, supporting the multi-component synergy principle of traditional Chinese medicine.

To validate these network pharmacology predictions, molecular docking analysis was performed between key active compounds and target proteins involved in mitochondrial dynamics and HIF-1α signaling. Kaempferol demonstrated significant binding affinity to HIF-1α with a docking score of −7.5 kcal/mol (Fig. 1D) and quercetin showed strong binding interactions with HIF-1α, achieving a docking score of −7.6 kcal/mol (Fig. 1E), supporting their roles in suppressing HIF-1α-mediated transcriptional activity. Similarly, quercetin showed excellent binding affinity to DRP1 with a docking score of −8.9 kcal/mol, forming multiple hydrogen bonds and hydrophobic interactions within the GTPase domain (Fig. 1F), and formononetin exhibited strong binding affinity to MFN1 with a docking score of −6.3 kcal/mol, forming stable interactions with the GTPase domain including hydrogen bonds with critical amino acid residues (Fig. 1G), suggesting their roles in modulating mitochondrial fission and fusion dynamics. These integrated network pharmacology and molecular docking results provide comprehensive evidence for M-SYFSF’s multi-target therapeutic approach, demonstrating that multiple active compounds can simultaneously target key proteins involved in mitochondrial dynamics (DRP1, MFN1) and metabolic reprogramming (HIF-1α), providing mechanistic insights into the nephroprotective effects observed in experimental studies.

### M-SYFSF ameliorates renal pathological changes and functional deterioration in diabetic kidney disease

**Fig. 2:**
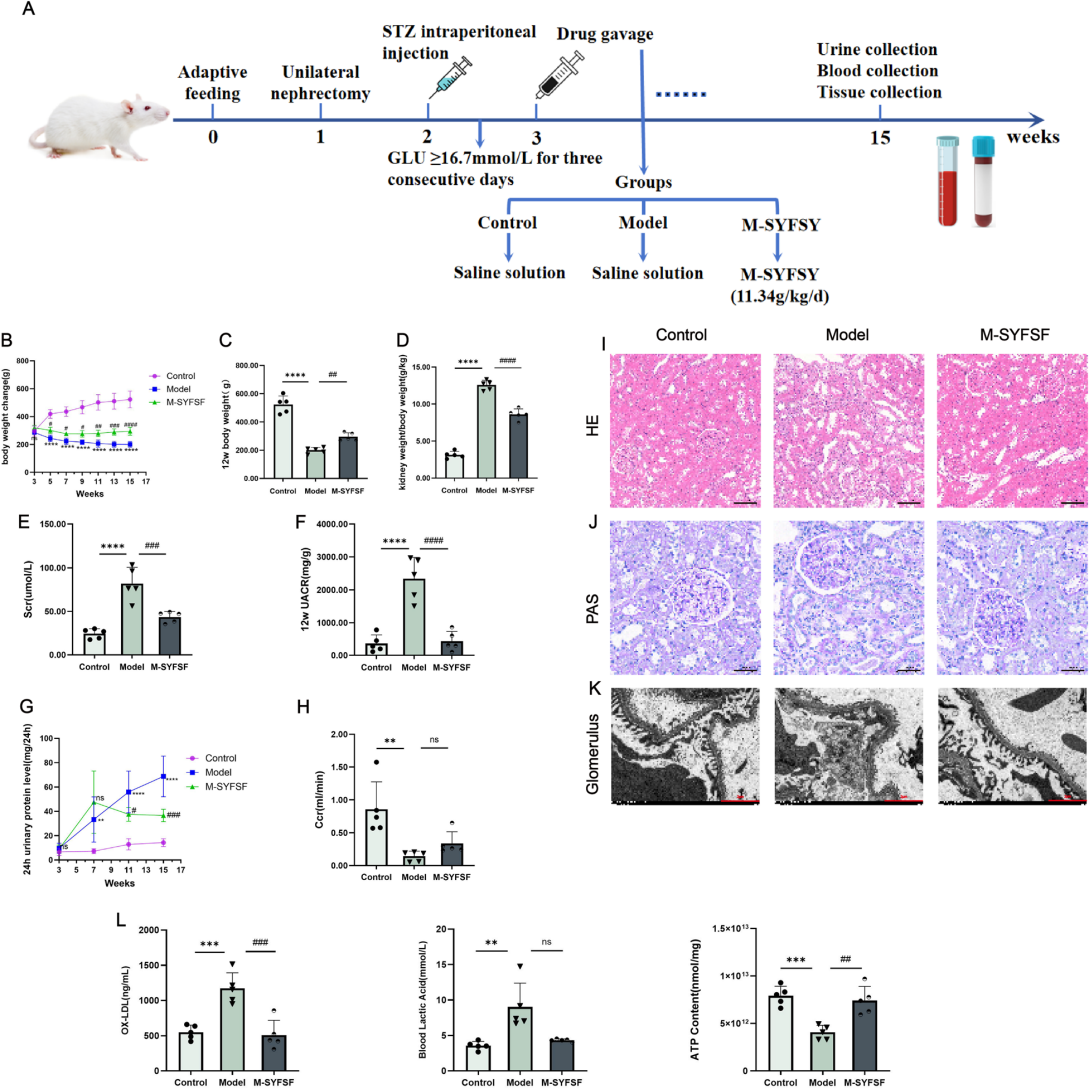
M-SYFSF ameliorates renal pathological changes and functional deterioration in DKD. **A** Schematic representation of the experimental animal procedure. **B** Body weight changes monitored weekly throughout the intervention period. **C**, **D** Blood glucose levels and kidney weight measurements. **E**–**H** Biochemical parameters including serum creatinine (Scr), 24 h urine protein (24 h-UTP), creatinine clearance (Ccr), and urinary albumin/creatinine ratio (UACR). **I** Representative H&E staining images showing glomerular and tubular morphology (scale bar = 50 μm). **J** PAS staining demonstrating glycogen deposits (scale bar = 50 μm). **K** Representative transmission electron microscopy images of glomerular ultrastructure. **L** Oxidative stress and metabolic markers including 8-OHdG levels, blood lactate, and ATP content. Data were analyzed by one-way ANOVA (mean ± SEM). n = 10. *p < 0.05, **p < 0.01, ***p < 0.001 compared with the control group; #p < 0.05, ##p < 0.01, ###p < 0.001 compared with the model group

We established a DKD model using unilateral nephrectomy followed by STZ-induced diabetes in rats and treated them with M-SYFSF. The experimental timeline shows the sequential procedures from adaptive feeding through sample collection is shown in Fig. 2A. Throughout the 15-week period, the model group showed significantly reduced weight gain compared to controls, while M-SYFSF treatment improved body weight recovery (Fig. 2B, C). Kidney weight significantly increased in the model group (*p* < 0.0001), while M-SYFSF treatment markedly reduced kidney weight (*p* < 0.001) (Fig. 2D). Biochemical analysis revealed that compared to controls, the model group exhibited significantly increased serum creatinine (*p* < 0.0001), and M-SYFSF treatment significantly reduced serum creatinine levels (*p* < 0.001) (Fig. 2E). Urinary albumin-to-creatinine ratio was markedly elevated in the model group (*p* < 0.0001), which was significantly reduced by M-SYFSF treatment (*p* < 0.001) (Fig. 2F). Additionally, 24-h urinary protein excretion was significantly elevated in the model group (*p* < 0.0001), which was markedly reduced by M-SYFSF treatment (*p* < 0.001) (Fig. 2G). Creatinine clearance was reduced in the model group (*p* < 0.01), with M-SYFSF treatment providing protective effects (Fig. 2H). Histological examination revealed that the model group exhibited characteristic DKD pathological features. H&E staining showed glomerular basement membrane thickening, mesangial expansion, and tubular damage (Fig. 2I). PAS staining demonstrated significant glycogen and protein deposits in glomerular and tubular structures (Fig. 2J). Transmission electron microscopy revealed podocyte foot process effacement and basement membrane irregularities in the model group (Fig. 2K). M-SYFSF treatment effectively ameliorated these pathological changes. Furthermore, 8-OHdG levels were elevated in the model group (*p* < 0.001), blood lactate increased (*p* < 0.01), and ATP content decreased (*p* < 0.001). M-SYFSF treatment significantly reduced 8-OHdG (*p* < 0.001) and partially restored ATP levels (*p* < 0.01) (Fig. 2L).

### M-SYFSF attenuates renal fibrosis in DKD

**Fig. 3:**
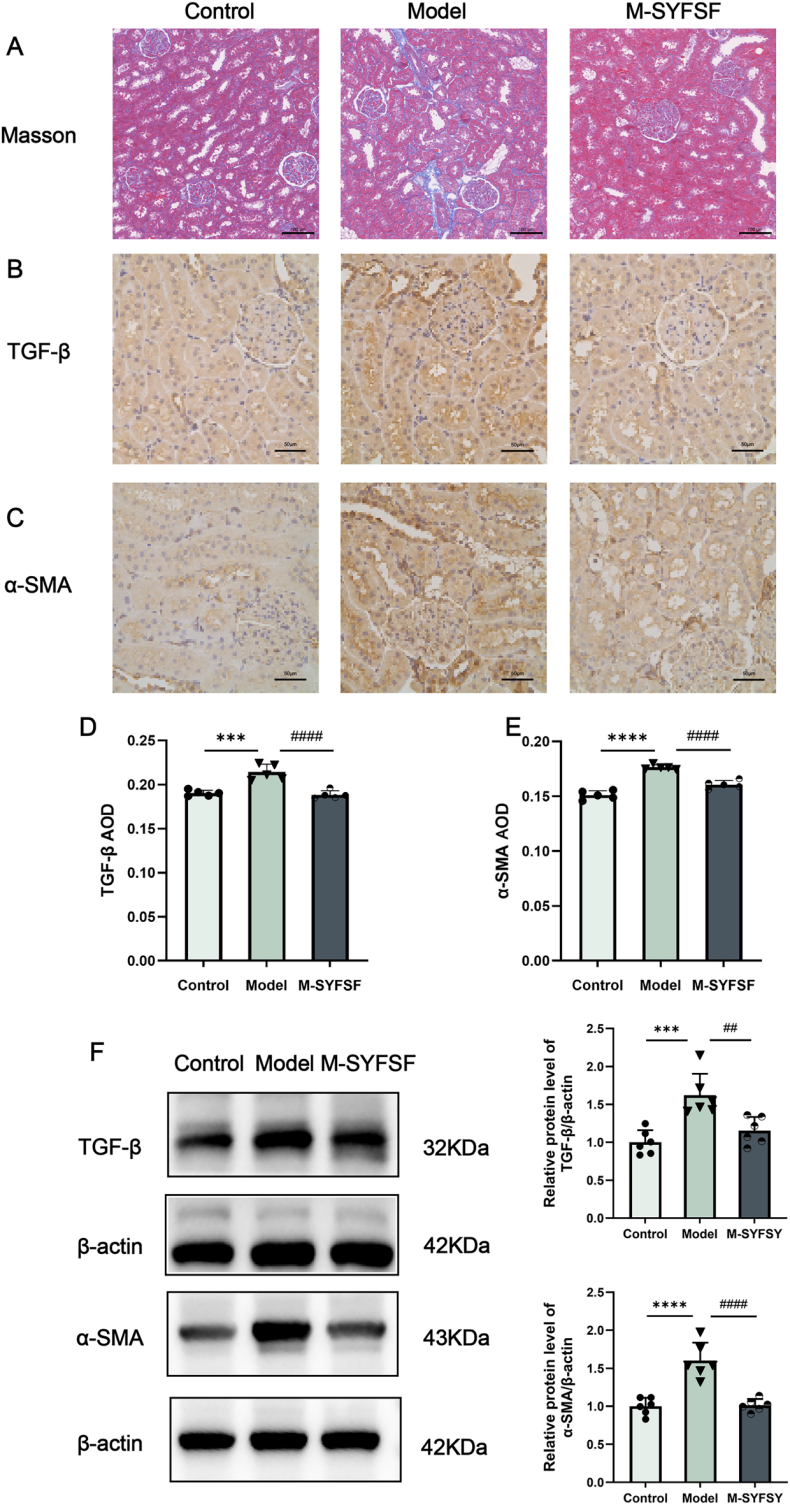
M-SYFSF attenuates renal fibrosis in DKD. **A** Representative Masson’s trichrome staining showing collagen deposition in renal tissues (scale bar = 50 μm). **B**, **C** Representative immunohistochemical staining of TGF-β and α-SMA proteins (scale bar = 50 μm). **D**, **E** Quantitative analysis of TGF-β and α-SMA expression levels by immunohistochemistry. **F** Western blot analysis and quantification of TGF-β and α-SMA protein levels in kidney tissues. Data were analyzed by one-way ANOVA (mean ± SEM). n = 6. *p < 0.05, ***p < 0.001, ****p < 0.0001 compared with the control group; #p < 0.05, ###p < 0.001, ####p < 0.0001 compared with the model group

To evaluate the anti-fibrotic effects of M-SYFSF, we examined renal fibrosis markers. Masson’s trichrome staining revealed minimal collagen deposition in controls, while the model group exhibited extensive interstitial fibrosis. M-SYFSF treatment markedly reduced collagen accumulation (Fig. 3A). Immunohistochemical analysis showed that TGF-β expression was significantly increased in the model group compared to controls, particularly in glomerular and tubular regions. M-SYFSF treatment notably reduced TGF-β expression (Fig. 3B). Similarly, α-SMA expression was substantially upregulated in the model group but effectively suppressed by M-SYFSF treatment (Fig. 3C). Quantitative analysis confirmed that TGF-β expression was significantly increased in the model group (*p* < 0.001), while M-SYFSF treatment significantly reduced TGF-β levels (*p* < 0.0001) (Fig. 3D). α-SMA expression was markedly elevated in the model group (*p* < 0.0001) and significantly decreased following M-SYFSF treatment (*p* < 0.0001) (Fig. 3E). Western blot analysis validated these findings. Compared to controls, the model group showed significant upregulation of TGF-β (*p* < 0.001) and α-SMA (*p* < 0.001). M-SYFSF treatment significantly reduced both TGF-β (*p* < 0.05) and α-SMA (*p* < 0.001) protein levels (Fig. 3F). Additionally, consistent with reduced fibrosis, M-SYFSF dose-dependently decreased serum IL-6 levels** (Supplementary Figure S2).**

### M-SYFSF ameliorates oxidative stress in diabetic kidney disease

**Fig. 4:**
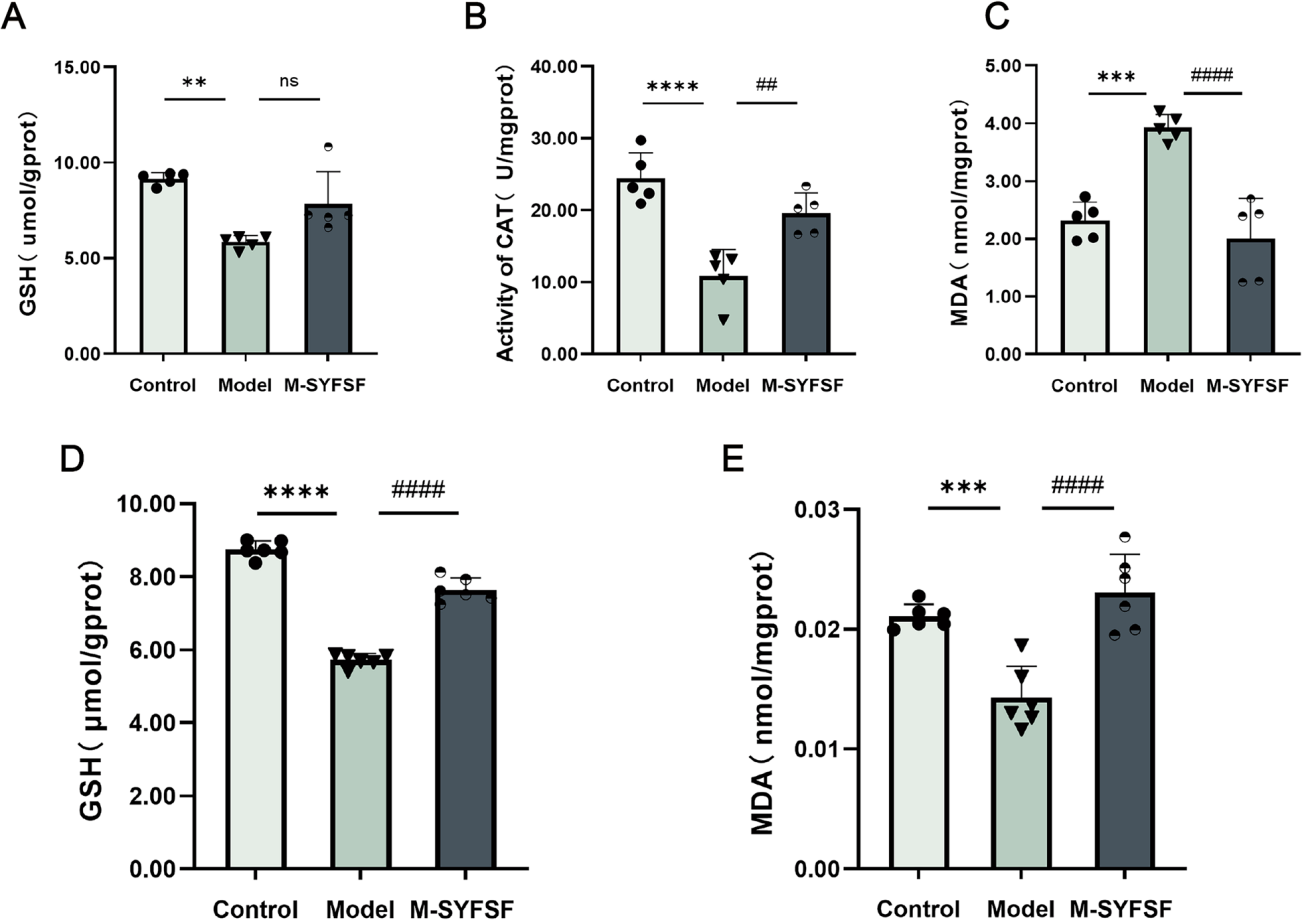
M-SYFSF ameliorates oxidative stress in DKD. **A**–**C** Antioxidant and oxidative stress markers in kidney tissues: GSH content, CAT activity, and MDA levels. **D**, **E** Oxidative stress markers in cultured renal tubular epithelial cells: GSH content and MDA levels. Data were analyzed by one-way ANOVA (mean ± SEM). n = 6. **p < 0.01, ***p < 0.001, ****p < 0.0001 compared with the control group; ##p < 0.01, ###p < 0.001, ####p < 0.0001 compared with the model group; ns = not significant

To investigate the antioxidant effects of M-SYFSF, we assessed oxidative stress markers in renal tissues and tubular epithelial cells. In kidney tissue, GSH levels were significantly decreased in the model group compared to controls (*p* < 0.01), while M-SYFSF treatment showed a trend toward restoration though not statistically significant (Fig. 4A). CAT activity was markedly reduced in the model group (*p* < 0.0001), and M-SYFSF treatment significantly restored CAT activity (*p* < 0.01) (Fig. 4B). MDA content, a marker of lipid peroxidation, was significantly elevated in the model group (*p* < 0.001), while M-SYFSF treatment significantly reduced MDA levels (*p* < 0.0001) (Fig. 4C).

In cultured renal tubular epithelial cells, similar patterns were observed. GSH content was significantly decreased in the model group (*p* < 0.0001), and M-SYFSF treatment significantly restored GSH levels (*p* < 0.0001) (Fig. 4D). MDA content was significantly elevated in the model group (*p* < 0.001), while M-SYFSF treatment significantly reduced MDA levels (*p* < 0.0001) (Fig. 4E). These results demonstrate that M-SYFSF effectively ameliorates oxidative stress by enhancing antioxidant capacity and reducing lipid peroxidation in diabetic kidney disease.

### M-SYFSF regulates glycolytic enzymes expression in diabetic kidney disease

**Fig. 5:**
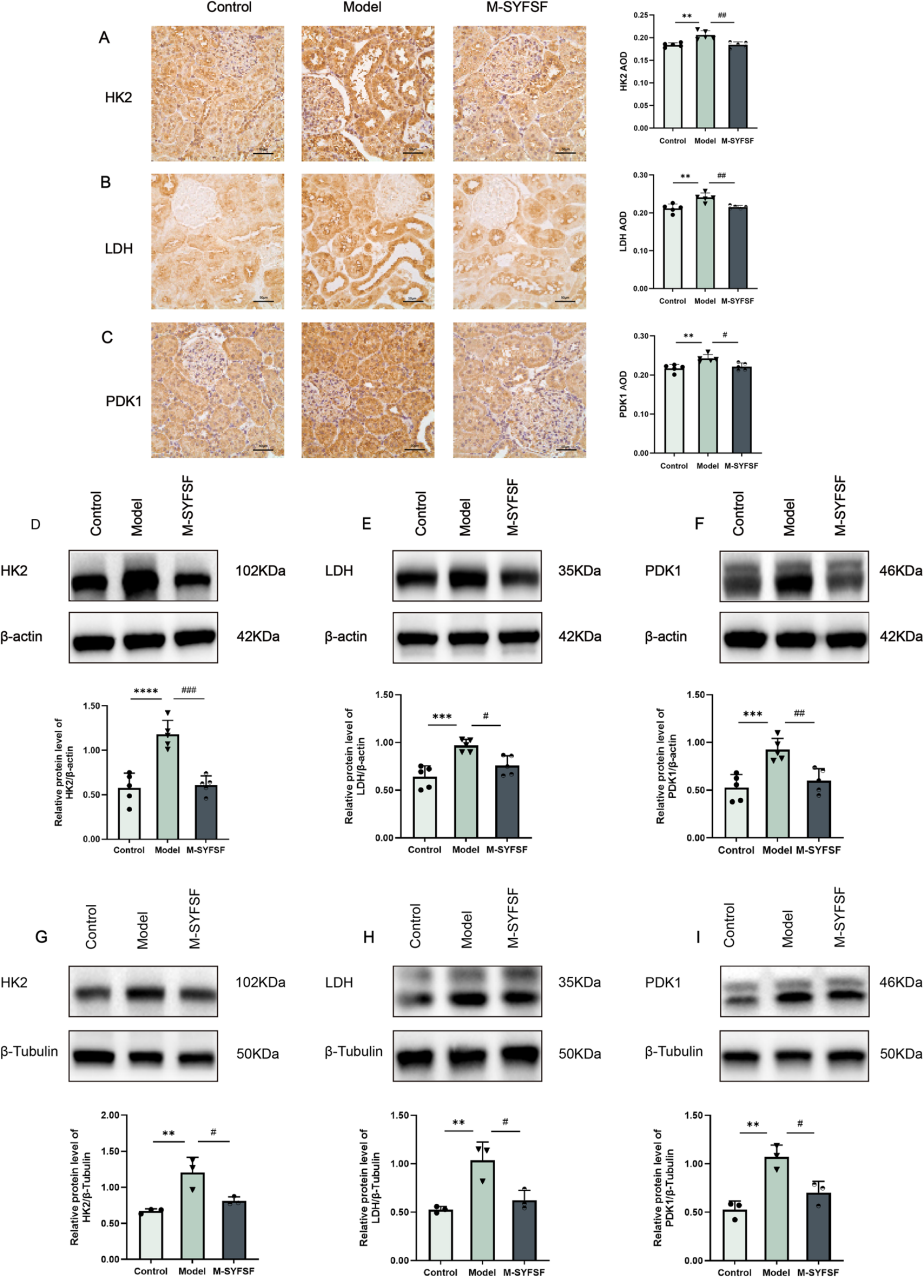
M-SYFSF regulates glycolytic enzyme expression in DKD. **A**–**C** Immunohistochemical analysis and quantification of HK2, LDH, and PDK1 expression in kidney tissues (scale bar = 50 μm). **D**–**F** Western blot analysis and quantification of glycolytic enzymes (HK2, LDH, PDK1) in kidney tissues. **G**–**I** Western blot analysis and quantification of glycolytic enzymes in cultured renal tubular epithelial cells. Data were analyzed by one-way ANOVA (mean ± SEM). n = 6. *p < 0.05, **p < 0.01, ***p < 0.001, ****p < 0.0001 compared with the control group; #p < 0.05, ##p < 0.01, ###p < 0.001 compared with the model group

To investigate the effects of M-SYFSF on glucose metabolism, we examined the expression of key glycolytic enzymes in renal tissues and tubular epithelial cells. Immunohistochemical analysis showed that HK2 expression was significantly increased in the model group compared to controls, while M-SYFSF treatment reduced HK2 expression (Fig. 5A). Similarly, LDH expression was markedly elevated in the model group and decreased following M-SYFSF treatment (Fig. 5B). PDK1 expression showed a similar pattern with increased expression in the model group and reduction after M-SYFSF treatment (Fig. 5C).

Western blot analysis in kidney tissues validated these findings. Compared to controls, the model group showed significant upregulation of HK2 (*p* < 0.0001), LDH (*p* < 0.001), and PDK1 (*p* < 0.001). M-SYFSY treatment significantly reduced HK2 (*p* < 0.001), LDH (*p* < 0.05), and PDK1 (*p* < 0.01) protein levels (Fig. 5D–F). In cultured renal tubular epithelial cells, similar results were observed. The model group exhibited significant increases in HK2 (*p* < 0.01), LDH (*p* < 0.01), and PDK1 (*p* < 0.01) expression, while M-SYFSF treatment significantly reduced all three glycolytic enzymes (Fig. 5G–I). These results demonstrate that M-SYFSF effectively modulates glycolytic metabolism by downregulating key glycolytic enzymes in diabetic kidney disease.

### M-SYFSF promotes metabolic shift from glycolysis to oxidative phosphorylation

**Fig. 6:**
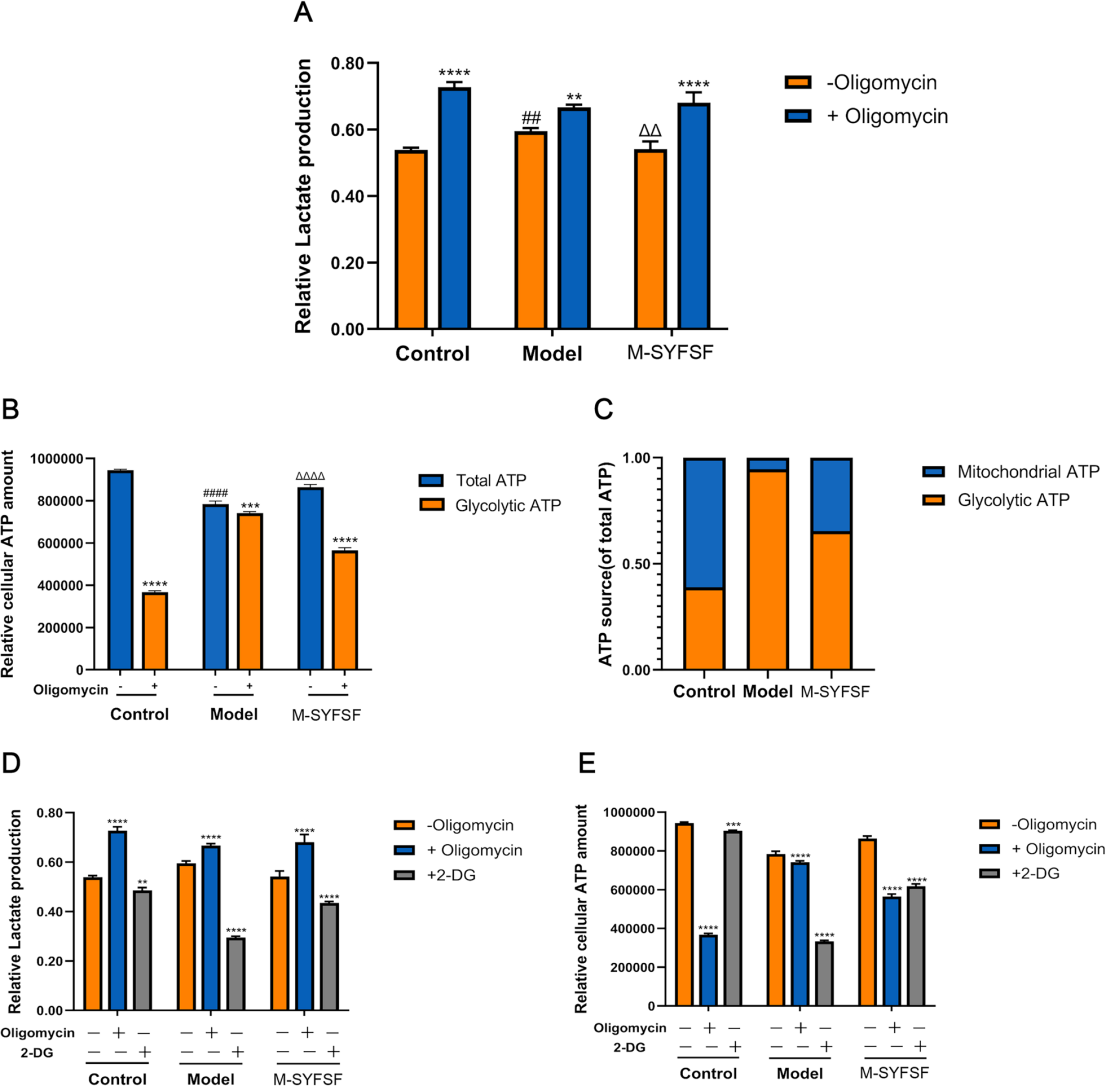
M-SYFSF promotes metabolic shift from glycolysis to oxidative phosphorylation. **A** Lactate production analysis with and without oligomycin treatment to assess glycolytic capacity. **B** ATP production analysis showing total ATP and mitochondrial ATP production capacity. **C** ATP source analysis demonstrating the relative contribution of mitochondrial oxidative phosphorylation versus glycolysis. **D**, **E** Metabolic dependence validation using 2-deoxy-D-glucose (2-DG) treatment showing lactate production and ATP levels. Data were analyzed by one-way ANOVA (mean ± SEM). n = 6. **p < 0.01, ***p < 0.001, ****p < 0.0001 within groups; ##p < 0.01 compared with the control group; ΔΔp < 0.01 compared with the model group

To investigate whether M-SYFSF promotes metabolic reprogramming from glycolysis to oxidative phosphorylation, we performed cellular bioenergetics analysis using oligomycin (an ATP synthase inhibitor) and 2-DG (a glycolysis inhibitor). Lactate production analysis showed that without oligomycin treatment, the model group had higher lactate levels than controls, while M-SYFSF treatment reduced lactate production. With oligomycin treatment, all groups showed increased lactate production due to compensatory glycolysis activation, with the difference between oligomycin-treated and untreated conditions representing glycolytic capacity (Fig. 6A). ATP production analysis revealed that without oligomycin, the model group had lower total ATP levels than controls, while M-SYFSF treatment increased ATP production. With oligomycin treatment, the difference between total ATP and remaining ATP represents mitochondrial ATP production capacity (Fig. 6B). The ATP source analysis demonstrated that controls relied more on mitochondrial oxidative phosphorylation, while the model group showed increased dependence on glycolytic ATP production. M-SYFSF treatment partially restored the balance toward oxidative phosphorylation (Fig. 6C).

To further validate metabolic dependence, we used 2-DG to inhibit glycolysis. The model group showed greater sensitivity to 2-DG treatment with more pronounced decreases in both lactate production (Fig. 6D) and ATP levels (Fig. 6E) compared to controls and M-SYFSF-treated groups, indicating higher glycolytic dependence. These results demonstrate that M-SYFSF promotes metabolic reprogramming from glycolysis toward oxidative phosphorylation in diabetic kidney disease.

### M-SYFSF improves mitochondrial structure and dynamics in diabetic kidney disease

**Fig. 7:**
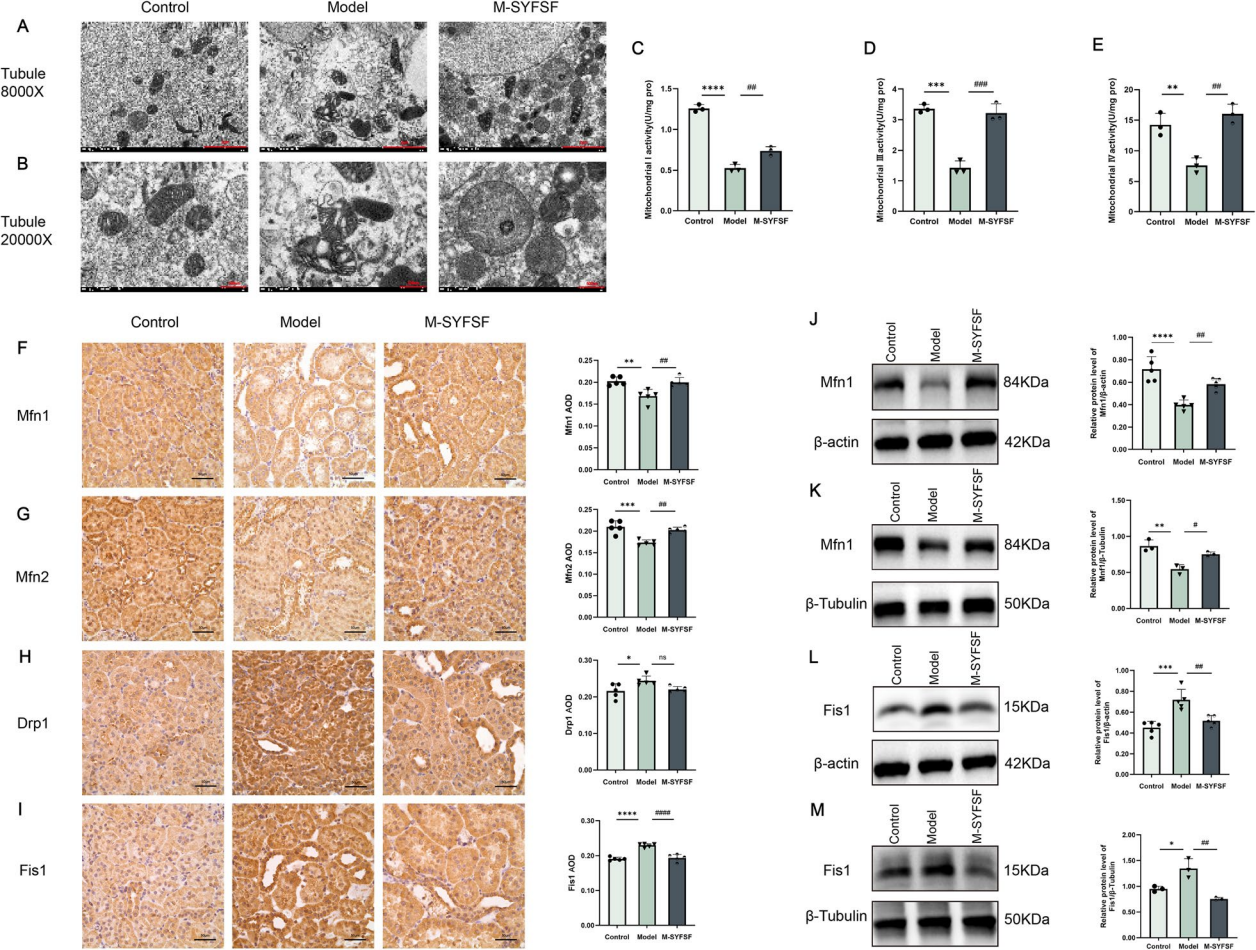
M-SYFSF improves mitochondrial structure and dynamics in DKD. **A**, **B** Representative transmission electron microscopy images of mitochondrial ultrastructure at different magnifications. Scale bars = 2 μm (8000 ×) and 500 nm (20,000 ×). **C**–**E** Quantitative analysis of mitochondrial morphological parameters and respiratory chain complex activities (I, III, and IV). **F**–**I** Representative immunohistochemical staining and quantitative analysis of mitochondrial dynamics proteins (Mfn1, Mfn2, Drp1, Fis1) (scale bar = 50 μm). **J**–**M** Western blot analysis and quantification of mitochondrial dynamics proteins in kidney tissues and cultured cells. Data were analyzed by one-way ANOVA (mean ± SEM). n = 6. *p < 0.05, **p < 0.01, ***p < 0.001, ****p < 0.0001 compared with the control group; #p < 0.05, ##p < 0.01, ###p < 0.001 compared with the model group

To investigate the effects of M-SYFSY on mitochondrial ultrastructure and dynamics, we performed transmission electron microscopy and examined mitochondrial fusion/fission proteins. Electron microscopy revealed that control group showed normal mitochondrial morphology with regular shape and intact cristae structure. The model group exhibited significant mitochondrial damage including swelling, cristae disruption, vacuolization, and reduced matrix density. M-SYFSY treatment markedly improved mitochondrial ultrastructure with restored cristae organization and reduced swelling (Fig. 7A, B).

To assess mitochondrial respiratory function, we measured the activities of mitochondrial respiratory chain complexes I, III, and IV in cultured renal tubular epithelial cells. The results showed that the model group significantly reduced the activities of mitochondrial complex I (*p* < 0.0001), complex III (*p* < 0.001), and complex IV (*p* < 0.01) compared to controls. M-SYFSY treatment significantly restored the activities of complex I (*p* < 0.01), complex III (*p* < 0.001), and complex IV (*p* < 0.01) (Fig. 7C–E).

To assess mitochondrial dynamics, we examined fusion and fission proteins by immunohistochemical staining and Western blot analysis. Immunohistochemical analysis showed that Mfn1 and Mfn2 expression were decreased in the model group compared to controls, while Drp1 and Fis1 expression were increased. M-SYFSY treatment restored fusion protein expression and reduced fission protein levels (Fig. 7F–I). Western blot analysis in kidney tissues validated these findings, showing that the model group had significant decreases in Mfn1 (*p* < 0.0001) and increases in Fis1 (*p* < 0.001), while M-SYFSY treatment significantly restored Mfn1 levels (*p* < 0.01) and reduced Fis1 expression (*p* < 0.01) (Fig. 7J–L). Similar patterns were observed in cultured renal tubular epithelial cells (Fig. 7K–M). These results demonstrate that M-SYFSY improves mitochondrial structure and promotes mitochondrial fusion while inhibiting excessive fission in DKD.

### M-SYFSF suppresses HIF-1α expression and nuclear translocation

**Fig. 8:**
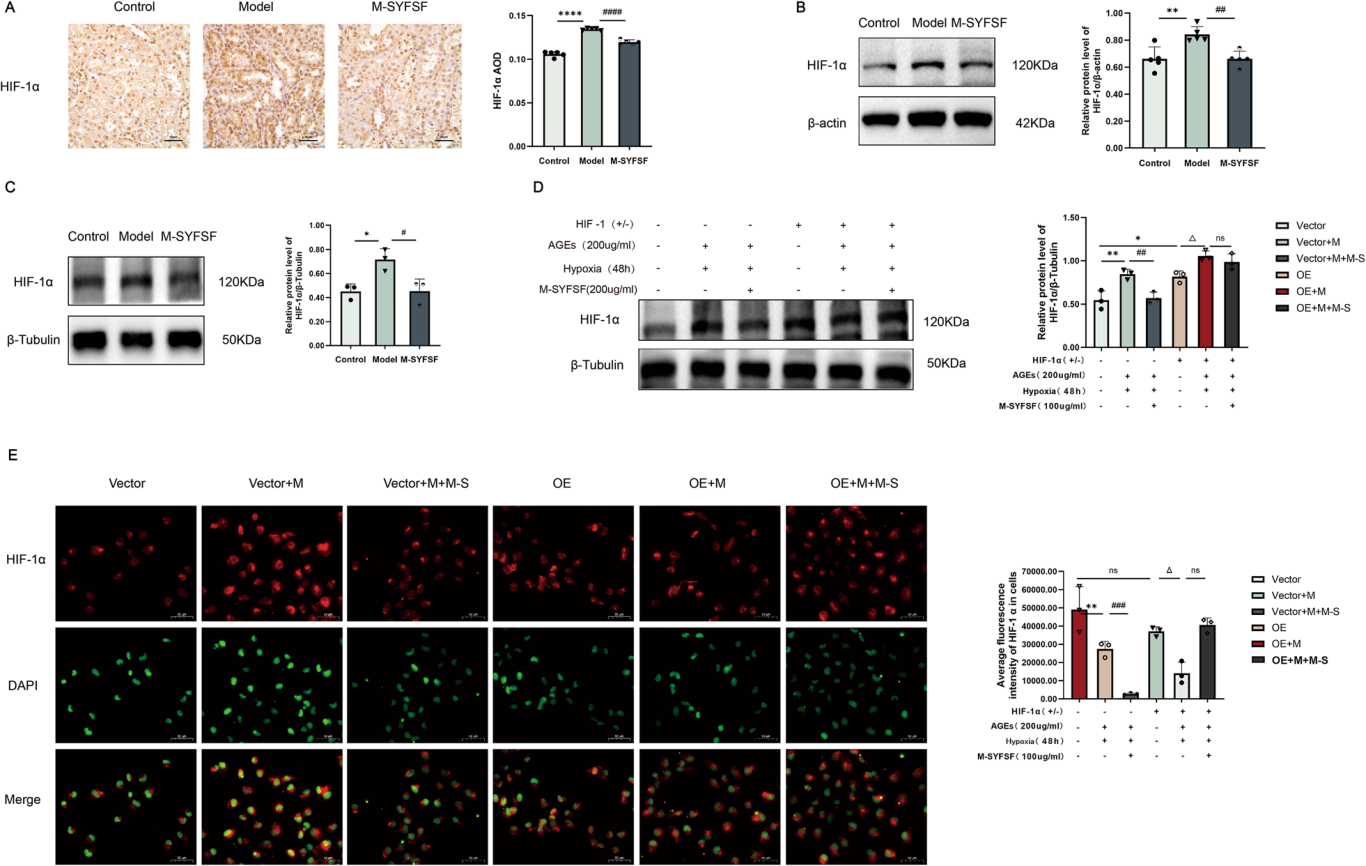
M-SYFSF suppresses HIF-1α expression and nuclear translocation. **A** Immunohistochemical analysis and quantification of HIF-1α expression in kidney tissues (scale bar = 50 μm). **B** Western blot analysis and quantification of HIF-1α protein levels in kidney tissues. **C** Western blot analysis and quantification of HIF-1α in cultured renal tubular epithelial cells. **D** HIF-1α overexpression validation in cell culture experiments. **E** Immunofluorescence analysis and quantification of HIF-1α subcellular localization and nuclear intensity (scale bar = 50 μm). Data were analyzed by one-way ANOVA (mean ± SEM). n = 6. *p < 0.05, **p < 0.01, ***p < 0.001, ****p < 0.0001 compared between groups as indicated

To investigate the effects of M-SYFSF on HIF-1α expression and subcellular localization, we performed immunohistochemical staining, Western blot analysis, and immunofluorescence microscopy. Immunohistochemical analysis showed that HIF-1α expression was significantly increased in the model group compared to controls, while M-SYFSF treatment markedly reduced HIF-1α expression (Fig. 8A).

Western blot analysis in kidney tissues confirmed these findings. The model group showed significant upregulation of HIF-1α protein expression (*p* < 0.01), while M-SYFSF treatment significantly reduced HIF-1α levels (*p* < 0.01) (Fig. 8B). Similar directional changes were observed in renal tubular epithelial cells, with the model group showing increased HIF-1α expression and M-SYFSF treatment reducing HIF-1α levels, though statistical significance varied between detection methods (Fig. 8C). To validate the role of HIF-1α in mediating M-SYFSF’s effects, we performed overexpression and knockdown experiments in renal tubular epithelial cells. HIF-1α overexpression (OE group) significantly increased HIF-1α protein levels compared to vector controls. AGEs treatment further enhanced HIF-1α expression, while M-SYFSF treatment reduced HIF-1α levels even in overexpressing cells (Fig. 8D).

Immunofluorescence analysis revealed that HIF-1α predominantly localized in the cytoplasm under normal conditions. AGEs stimulation promoted HIF-1α nuclear translocation, as evidenced by increased nuclear fluorescence intensity (*p* < 0.01). M-SYFSF treatment significantly inhibited HIF-1α nuclear translocation (*p* < 0.001), maintaining HIF-1α in the cytoplasm (Fig. 8E). These results demonstrate that M-SYFSF suppresses HIF-1α expression and prevents its nuclear translocation in diabetic kidney disease.

### M-SYFSY inhibits HIF-1α-mediated glycolytic reprogramming

**Fig. 9:**
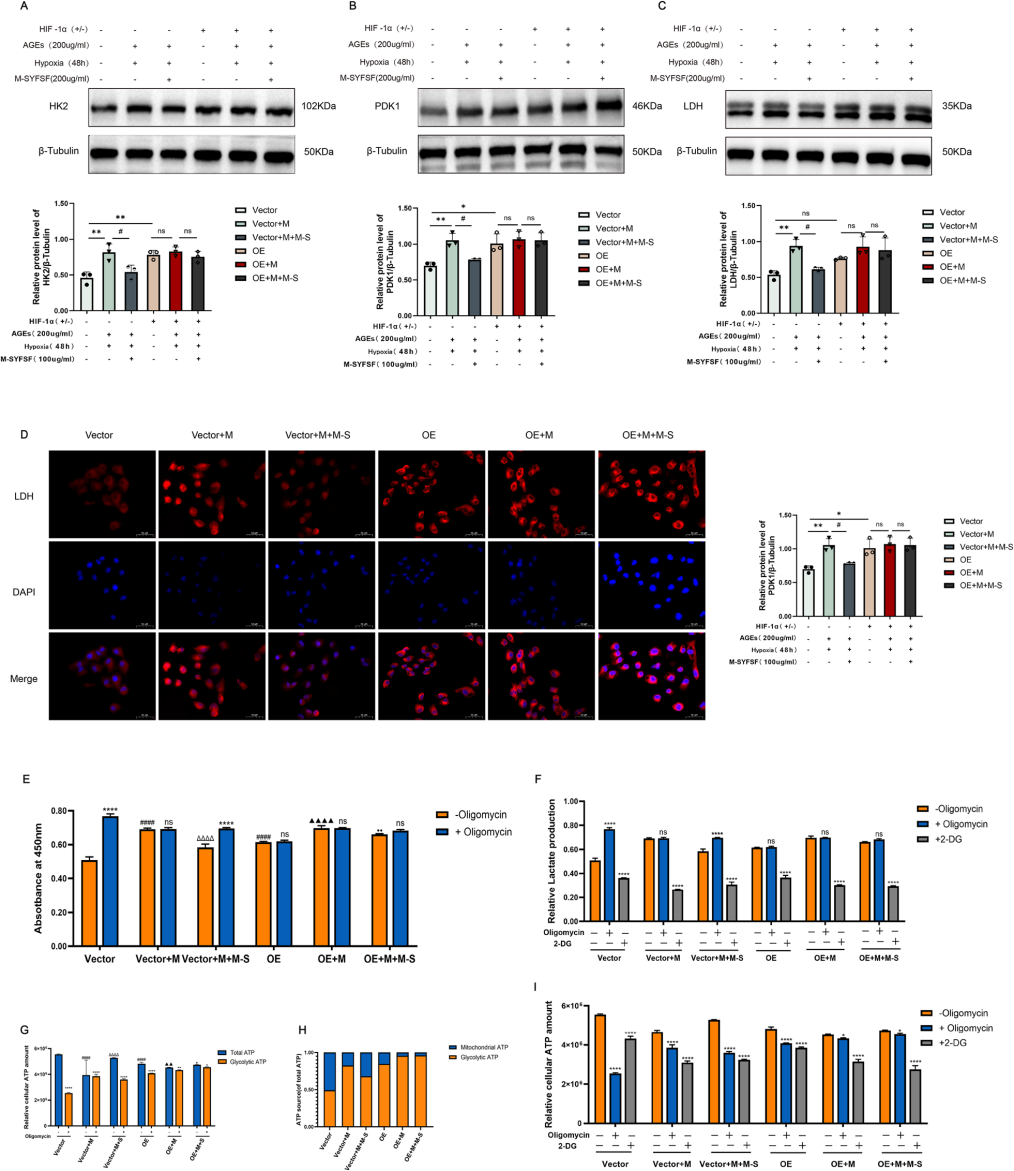
M-SYFSF inhibits HIF-1α-mediated glycolytic reprogramming. **A**–**C** Western blot analysis and quantification of glycolytic enzymes (HK2, PDK1, LDH) in HIF-1α overexpression experiments. **D** Immunofluorescence analysis and quantification of LDH expression (scale bar = 50 μm). **E**–**I** Cellular bioenergetics analysis including glycolytic capacity measurement, ATP production analysis, and ATP source determination using oligomycin and 2-DG treatments. Experimental groups: Vector, Vector + M (Model), Vector + M + M-S (Model + M-SYFSF), OE (Overexpression), OE + M, OE + M + M-S. Data were analyzed by one-way ANOVA (mean ± SEM). n = 6. *p < 0.05, **p < 0.01, ***p < 0.001, ****p < 0.0001 compared between groups as indicated

To investigate whether M-SYFSY’s effects on glycolytic metabolism are mediated through HIF-1α, we performed gain- and loss-of-function experiments in cultured renal tubular epithelial cells. Western blot analysis showed that HIF-1α overexpression significantly increased the expression of glycolytic enzymes HK2, PDK1, and LDH compared to vector controls. AGEs treatment further enhanced these effects, while M-SYFSY treatment reduced glycolytic enzyme expression even in HIF-1α overexpressing cells (Fig. 9A–C).

Immunofluorescence analysis revealed that HIF-1α overexpression promoted LDH expression and cellular localization. AGEs stimulation enhanced LDH fluorescence intensity, while M-SYFSY treatment effectively suppressed LDH expression regardless of HIF-1α status (Fig. 9D).

To assess functional metabolic changes, we performed cellular bioenergetics analysis using oligomycin and 2-DG treatments. HIF-1α overexpression significantly increased glycolytic capacity as measured by extracellular acidification rate (ECAR) following oligomycin treatment (*p* < 0.0001). AGEs stimulation further enhanced glycolytic flux, while M-SYFSY treatment significantly reduced glycolytic activity even in HIF-1α overexpressing cells (Fig. 9E, F). ATP production analysis demonstrated that HIF-1α overexpression promoted glycolytic ATP generation at the expense of mitochondrial ATP production. The ratio of mitochondrial to glycolytic ATP was significantly reduced in HIF-1α overexpressing cells (Fig. 9G, H). ATP source analysis confirmed that HIF-1α overexpression shifted cellular energy metabolism toward glycolysis, while M-SYFSY treatment promoted oxidative phosphorylation even under HIF-1α overexpression conditions (Fig. 9I).

### M-SYFSY modulates HIF-1α-mediated mitochondrial dynamics

**Fig. 10:**
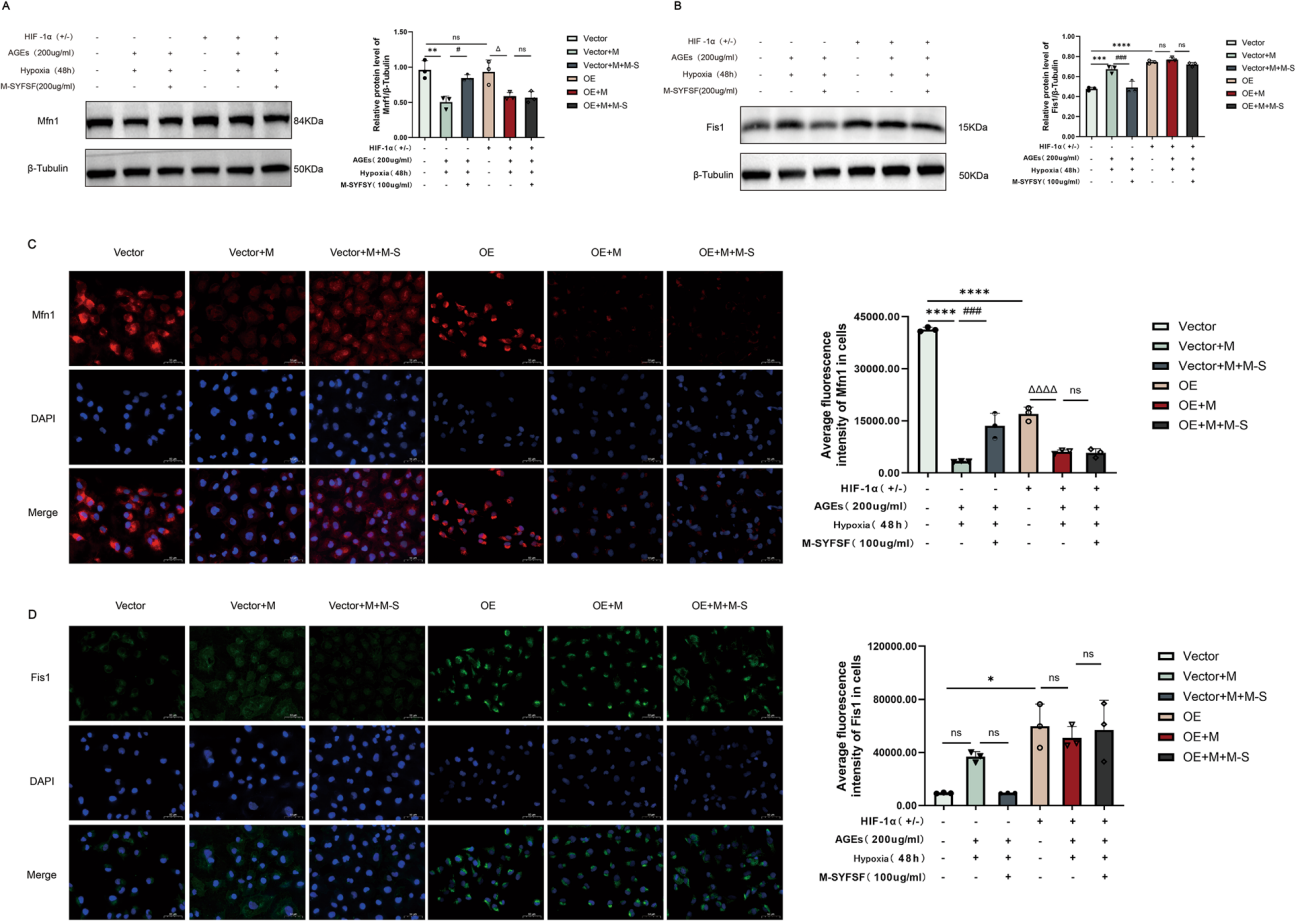
M-SYFSF modulates HIF-1α-mediated mitochondrial dynamics. **A**, **B** Western blot analysis and quantification of mitochondrial fusion protein Mfn1 and fission protein Fis1 in HIF-1α overexpression experiments. **C**, **D** Immunofluorescence analysis and quantification of Mfn1 and Fis1 expression (scale bar = 50 μm). Experimental groups: Vector, Vector + M, Vector + M + M-S, OE, OE + M, and OE + M + M-S. Data were analyzed by one-way ANOVA (mean ± SEM). n = 6. *p < 0.05, **p < 0.01, ***p < 0.001, ****p < 0.0001 compared between groups as indicated; ns = not significant

To investigate whether M-SYFSY’s effects on mitochondrial dynamics are mediated through HIF-1α, we examined mitochondrial fusion and fission proteins in HIF-1α overexpression and knockdown experiments. Western blot analysis showed that HIF-1α overexpression significantly decreased Mfn1 expression compared to vector controls (*p* < 0.01). AGEs treatment further reduced Mfn1 levels, while M-SYFSF treatment restored Mfn1 expression even in HIF-1α overexpressing cells, with consistent directional changes observed across different experimental conditions (Fig. 10A). Conversely, HIF-1α overexpression increased Fis1 expression (*p* < 0.0001), and this effect was enhanced by AGEs stimulation. M-SYFSY treatment significantly reduced Fis1 levels regardless of HIF-1α status (Fig. 10B).

Immunofluorescence analysis revealed that HIF-1α overexpression dramatically reduced Mfn1 fluorescence intensity, indicating decreased mitochondrial fusion protein expression. AGEs treatment further suppressed Mfn1 expression, while M-SYFSY treatment effectively restored Mfn1 levels even under HIF-1α overexpression conditions (Fig. 10C). In contrast, Fis1 expression was barely detectable in vector controls but was markedly increased following HIF-1α overexpression. AGEs stimulation enhanced Fis1 expression, while M-SYFSY treatment reduced Fis1 levels (Fig. 10D).

These results demonstrate that M-SYFSY modulates mitochondrial dynamics by counteracting HIF-1α-mediated suppression of mitochondrial fusion and promotion of mitochondrial fission. M-SYFSY treatment promotes mitochondrial fusion while inhibiting excessive fission, contributing to improved mitochondrial function and cellular energy metabolism in DKD.

## Discussion

**Fig. 11:**
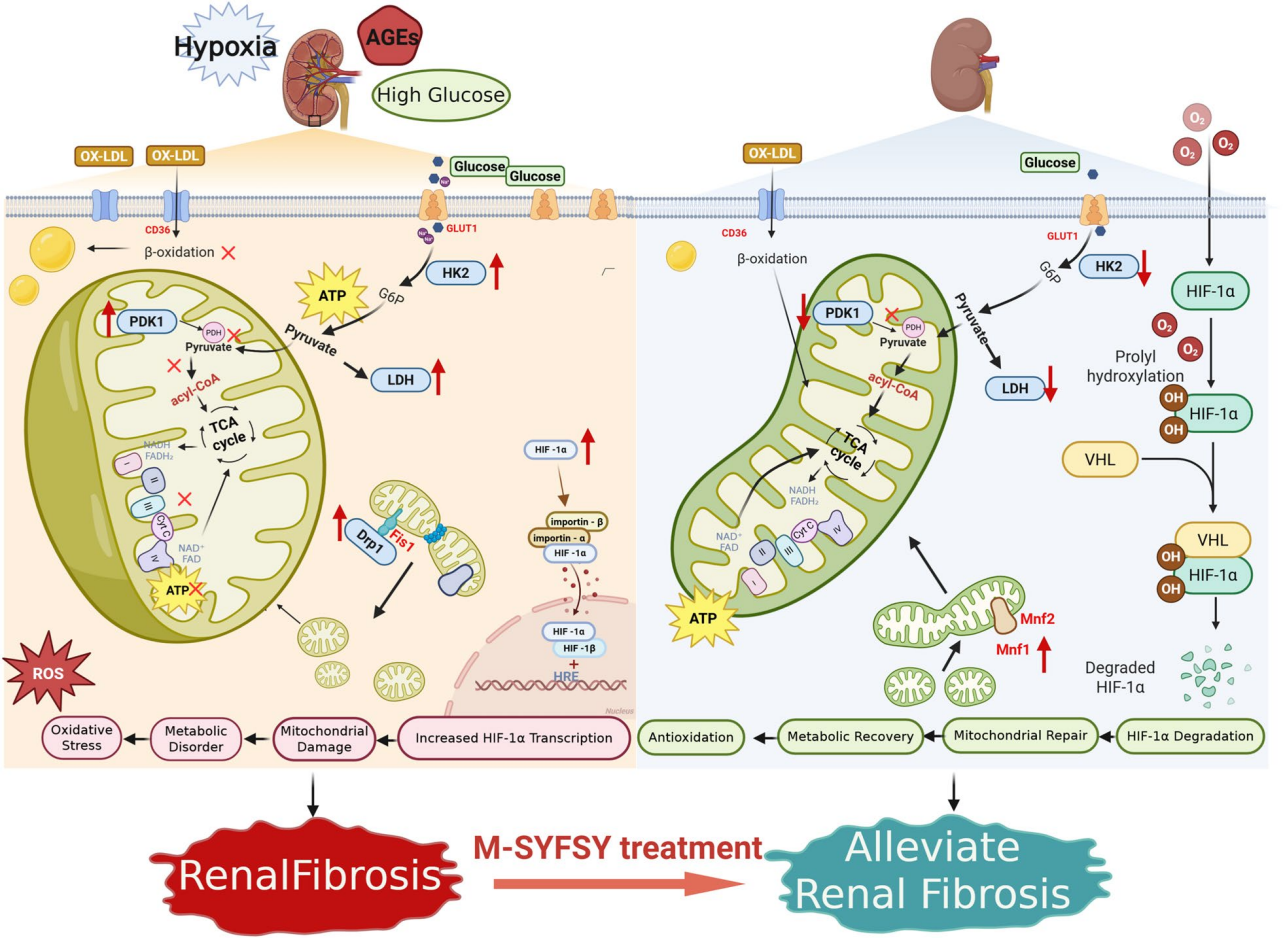
Proposed mechanism of M-SYFSF therapeutic action in DKD

This study demonstrates that M-SYFSY exerts significant nephroprotective effects in diabetic kidney disease through the modulation of HIF-1α-mediated metabolic reprogramming. Our findings establish HIF-1α as a central therapeutic target and provide novel mechanistic insights into how targeting this pathway can restore metabolic homeostasis and prevent kidney injury progression. Figure 11 provides a comprehensive mechanistic model illustrating how M-SYFSF modulates HIF-1α-mediated metabolic reprogramming to ameliorate diabetic kidney disease.

### HIF-1α as a central mediator in DKD

The role of HIF-1α in diabetic kidney disease has been increasingly recognized as both complex and context-dependent. Recent studies have shown that HIF-1α activation exhibits cell-specific effects in the diabetic kidney, with elevated HIF activity in glomerular cells promoting glomerulosclerosis and albuminuria, while tubular HIF activity is often suppressed, leading to mitochondrial dysfunction and tubulointerstitial fibrosis [15]. Our findings align with recent evidence demonstrating that HIF-1α serves as a therapeutic target, as SGLT2 inhibitors have been shown to protect diabetic kidneys by targeting HIF-1α protein and inhibiting hypoxia-induced HIF-1α accumulation [12].

The dual role of HIF-1α in kidney disease is further supported by studies showing that tubular-specific deletion of HIF-1α in diabetic mice exacerbates mitochondrial dysfunction and ROS accumulation, leading to tubular damage [27], while HIF-1α deficiency accelerates pathological changes in the early stages of DKD, particularly affecting podocyte survival and glomerular injury [29]. However, our study focuses on the pathological overactivation of HIF-1α under diabetic conditions, where excessive HIF-1α activation drives metabolic reprogramming toward glycolysis and contributes to disease progression.

Importantly, our HIF-1α overexpression experiments revealed that M-SYFSF treatment partially but not completely reversed HIF-1α-induced metabolic changes, indicating that while HIF-1α is a primary and critical mediator, additional HIF-1α-independent mechanisms may also contribute to M-SYFSF’s therapeutic efficacy. Possible additional mechanisms include: (1) regulation of other metabolic transcription factors such as Nrf2 or PGC-1α; (2) post-translational modulation of HIF-1α activity; (3) direct effects on metabolic enzyme activities; or (4) modulation of upstream signaling pathways (PI3K/Akt, AMPK). These possibilities warrant future investigation. This multi-pathway nature provides therapeutic advantages: multiple mechanisms ensure efficacy even when individual pathways are dysregulated, reduce compensatory resistance, and provide balanced metabolic regulation. This aligns with Traditional Chinese Medicine principles of multi-component, multi-target intervention and suggests clinical advantages for complex chronic diseases like DKD. While complete mechanistic dissection requires additional studies, our comprehensive data establish HIF-1α as a central mechanistic node in M-SYFSF’s therapeutic action.

### Metabolic reprogramming as a therapeutic target

The concept of metabolic reprogramming, originally described as the Warburg effect in cancer cells, has gained significant attention in kidney disease research. Metabolic reprogramming involves a shift from mitochondrial oxidative phosphorylation to glycolysis and its side branches, and is thought to play a critical role in the development and progression of DKD [8]. Recent studies indicate that mitochondrial metabolic reprogramming significantly influences the pathophysiological progression of DKD, with alterations in kidney metabolism leading to abnormal expression of signaling molecules and activation of pathways that induce oxidative stress-related cellular damage and renal fibrosis [17]. Our findings demonstrate that M-SYFSY effectively reverses pathological metabolic reprogramming by inhibiting HIF-1α-mediated transcriptional activation of key glycolytic enzymes including HK2, PDK1, and LDH. This is consistent with recent evidence showing that HIF-1α target genes include PAI-1, VEGF, GLUT1, HK2, and PKM, and that therapeutic interventions can effectively modulate these pathways [12].

### Mitochondrial dysfunction and dynamics in DKD

Mitochondrial dysfunction represents a central pathogenic mechanism in DKD. DKD is characterized by mitochondrial dysfunction, with the kidney ranking second only to the heart in terms of molecular oxygen consumption, highlighting its significant energy requirements [30]. Our study reveals that M-SYFSY protects mitochondrial structure and function while promoting mitochondrial fusion through upregulation of Mfn1 and Mfn2 and downregulation of Drp1 and Fis1.

Alteration of mitochondrial dynamics toward mitochondrial fission concurrent with mitochondrial dysfunction is characteristic of diabetic kidney disease, and these perturbations may be responsible for the residual risk of end-stage renal disease in diabetic kidney disease patients [31]. Recent studies have shown that mitochondrial dynamics proteins represent emerging therapeutic targets [32], with several compounds demonstrating the ability to modulate mitochondrial fusion and fission in diabetic nephropathy models.

The importance of mitochondrial dynamics is further emphasized by studies showing that an accumulation of fragmented mitochondria is found in the renal cortex in both humans and animals with diabetic nephropathy, suggesting that mitochondrial clearance mechanisms may be impaired, and that impairment in the mitophagy system leads to accelerated progression of renal pathology [15]. A recent study using Drosophila models demonstrated that promoting mitochondrial dynamics by inhibiting the PINK1-PRKN pathway can relieve diabetic nephropathy [33].

### Oxidative stress and antioxidant mechanisms

The antioxidant properties of M-SYFSY represent a crucial therapeutic dimension that complements its metabolic effects. Our findings demonstrate significant reductions in lipid peroxidation markers and enhancement of antioxidant enzyme activities. This is consistent with recent evidence showing that oxidative stress plays a crucial role in diabetic kidney disease progression, and that enhancing mitochondrial function can attenuate inflammation and fibrosis, thereby reducing the severity of diabetic complications [34].

Studies have shown that HIF-1α can exert protective effects against tubular injury in diabetic nephropathy via HO-1-mediated control of mitochondrial dynamics [27], suggesting complex interactions between HIF-1α signaling, oxidative stress responses, and mitochondrial function. Our study adds to this understanding by demonstrating that controlled inhibition of pathologically activated HIF-1α can restore antioxidant capacity while preserving mitochondrial function. The mutual regulation between redox and hypoxia-inducible factors in cardiovascular and renal complications of diabetes has been recently reviewed [35]. While direct ROS measurement would provide more direct evidence, our assessment through MDA levels and antioxidant enzyme activities, combined with improved mitochondrial function, strongly supports M-SYFSF’s antioxidant effects in DKD. Meanwhile, The reduction of IL-6 demonstrates M-SYFSF’s anti-inflammatory effects, as IL-6 contributes to DKD through mesangial proliferation and ECM accumulation, potentially linking inflammation to fibrosis progression.

### Network pharmacology and molecular docking validation of multi-target mechanisms

Our integrated network pharmacology and molecular docking analyses provide compelling evidence for M-SYFSF’s multi-target therapeutic approach. The identification of 570 common targets between M-SYFSF compounds and DKD demonstrates comprehensive therapeutic coverage, consistent with traditional Chinese medicine’s multi-component synergistic approach. The prominent enrichment of HIF-1 signaling pathway provides strong bioinformatic support for our experimental findings on HIF-1α-mediated metabolic reprogramming, while additional pathways including AGE-RAGE signaling and complement cascades suggest M-SYFSF addresses multiple aspects of DKD pathophysiology simultaneously.

The molecular docking results provide structural basis for therapeutic interactions. Quercetin's strong binding affinity to DRP1 (−8.9 kcal/mol) offers mechanistic insight into reduced mitochondrial fission, while formononetin’s binding to MFN1 (−6.3 kcal/mol) supports enhanced mitochondrial fusion observed experimentally. These docking scores exceed the significance threshold (−5.0 kcal/mol), indicating genuine therapeutic potential. The binding of multiple compounds to HIF-1α validates this transcription factor as a central therapeutic target and explains how M-SYFSF modulates HIF-1α-mediated metabolic reprogramming through complementary mechanisms.

This computational-experimental integration represents a valuable paradigm for traditional Chinese medicine research, enabling rational identification of active components while maintaining holistic treatment philosophy. The convergence of network pharmacology predictions with experimental validation strengthens confidence in M-SYFSF’s therapeutic mechanisms and supports clinical translation potential.

### Novel therapeutic approach and mechanistic innovation

M-SYFSY represents a mechanistically novel therapeutic approach that differs from current standard therapies. While existing treatments primarily focus on hemodynamic modifications through ACE inhibitors, ARBs, and SGLT2 inhibitors, M-SYFSY directly targets the metabolic dysfunction underlying diabetic kidney disease pathogenesis. The kidneys are high-energy-consuming organs that require a large amount of energy to remove waste from blood, reabsorb nutrients, balance electrolytes and fluids, maintain acid–base homeostasis, and regulate blood pressure [17], making metabolic dysfunction a particularly relevant therapeutic target.

The multi-target approach of M-SYFSY, simultaneously addressing HIF-1α activation, metabolic reprogramming, mitochondrial dysfunction, and oxidative stress, offers potential advantages over single-pathway interventions. Current investigations reveal the crucial role of targeting metabolic reprogramming pathways for the amelioration of kidney disease [36], with emphasis on therapies targeting these pathways. Recent clinical studies demonstrate that SGLT2 inhibitors restore metabolic perturbations in proximal tubular cells and reduce inflammatory signaling [37].

### Study limitations and future directions

Several limitations of this study warrant consideration. The research was conducted exclusively in male rats, and sex-specific differences in diabetic kidney disease progression and treatment responses require investigation. Recent bioinformatics and machine learning approaches have identified metabolic reprogramming-related genes as potential diagnostic biomarkers for DKD [38], suggesting opportunities for personalized therapeutic approaches that our study did not explore.

The optimal dosing regimen, long-term safety profile, and potential drug interactions of M-SYFSY need further evaluation. Additionally, while our mechanistic studies focused on HIF-1α, other transcription factors and signaling pathways may contribute to diabetic kidney pathology. The development of HIF-prolyl hydroxylase inhibitors for treating renal anemia provides insights into the complex regulation of HIF pathways [39], suggesting that precise modulation rather than complete inhibition may be optimal.

Future research directions should include clinical translation studies to evaluate M-SYFSY's safety and efficacy in human diabetic kidney disease. Understanding the modulation of mitochondrial function and immunometabolism represents a critical strategy for decelerating diabetic kidney disease progression [7], suggesting that M-SYFSY’s effects on immune cell metabolism warrant investigation. Dose–response studies, pharmacokinetic analyses, and investigation of combination therapies with existing nephroprotective agents will be essential for clinical development.

### Study limitations and future directions

This study has several limitations that suggest important directions for future research. First, regarding mechanistic understanding, we did not perform chromatin immunoprecipitation (ChIP) experiments to directly demonstrate HIF-1α binding to target gene promoters. However, coordinated downregulation of multiple HIF-1α targets (HK2, LDH, PDK1), reduced nuclear translocation, and comprehensive functional validation through loss- and gain-of-function experiments strongly support HIF-1α-mediated transcriptional regulation. Future ChIP studies would provide direct binding evidence and identify the complete spectrum of transcriptional targets. Additionally, while HIF-1α represents a central therapeutic target, other transcription factors and pathways may contribute to M-SYFSF’s multi-target effects. Second, experimental design considerations include: (1) use of only male rats sex-specific differences in DKD require investigation; (2) extract storage with repeated freeze–thaw cycles—while consistent procedures and robust results suggest adequate stability, single-use aliquots would be preferable; and (3) absence of routine postoperative analgesia to avoid confounding renal effects—we implemented enhanced monitoring and supportive care with successful outcomes, though alternative analgesic approaches warrant exploration. Third, while we demonstrated anti-inflammatory effects through IL-6 measurement, future studies should include comprehensive cytokine profiling (IL-1β, TNF-α, MCP-1) to fully characterize M-SYFSF's immunomodulatory effects in DKD. Fourth, we did not perform direct ROS quantification, future studies should include direct ROS measurement to provide definitive evidence for temporal and spatial dynamics of ROS reduction by M-SYFSF. Fifth, clinical translation is essential. Future studies should evaluate M-SYFSF’s safety and efficacy in human DKD through dose–response studies, pharmacokinetic analyses, and investigation of combination therapies with existing nephroprotective agents. These efforts will be critical for advancing M-SYFSF toward clinical application.

## Conclusion

In conclusion, M-SYFSY represents a promising therapeutic candidate for diabetic kidney disease that addresses fundamental metabolic dysfunction underlying disease pathogenesis. By targeting HIF-1α-mediated metabolic reprogramming primarily, while potentially engaging additional therapeutic pathways consistent with Traditional Chinese Medicine’s multi-target approach, M-SYFSY offers a mechanistically novel approach to nephroprotection that complements existing therapies. The comprehensive efficacy demonstrated across multiple pathological domains, combined with the growing recognition of metabolic reprogramming as a central mechanism in kidney disease, positions M-SYFSY as a potential cornerstone therapy for diabetic kidney disease management. However, careful clinical translation will be required to realize this therapeutic potential and improve outcomes for patients with DKD.

## Supplementary Information

Supplementary Material 1

## Data Availability

The data supporting the findings of this study are available within the article and its supplementary materials. Additional data are available from the corresponding author upon reasonable request.

## Abbreviations

ACE: Angiotensin-converting enzyme
AGEs: Advanced glycation end products
ARB: Angiotensin receptor blocker
ATP: Adenosine triphosphate
CAT: Catalase
CCK-8: Cell counting kit-8
CKD: Chronic kidney disease
DKD: Diabetic kidney disease
DMSO: Dimethyl sulfoxide
Drp1: Dynamin related protein 1
ESRD: End-stage renal disease
Fis1: Fission 1
GSH: Glutathione
H&E: Hematoxylin and eosin
HIF-1α: Hypoxia-inducible factor-1α
HK-2: Human proximal tubular epithelial cells
HK2: Hexokinase 2
LDH: Lactate dehydrogenase
MDA: Malondialdehyde
Mfn1/Mfn2: Mitofusin ½
M-SYFSF: Modified Shen-Yan-Fang-Shuai formula
OB: Oral bioavailability
PAS: Periodic acid-schiff
PDB: Protein data bank
PDK1: Pyruvate dehydrogenase kinase 1
RT-PCR: Reverse transcription-polymerase chain reaction
SGLT2: Sodium-glucose cotransporter 2
STZ: Streptozotocin
UACR: Urinary albumin-to-creatinine ratio
2-DG: 2-Deoxy-d-glucose
8-OHdG: 8-Hydroxy-2’-deoxyguanosine
α-SMA: α-Smooth Muscle Actin
