# Supplementary material for: Modified Shen-Yan-Fang-Shuai formula attenuates diabetic kidney disease progression via regulation of HIF-1α-mediated mitochondrial energy metabolism
Source: Chin Med. 2026 Jan 9;21:23. doi: 10.1186/s13020-025-01298-5 (PMC12784558; doi:10.1186/s13020-025-01298-5)

**Supplementary Figure S1. Experimental design and flow chart of animal**

**allocation and treatment protocol.**

**
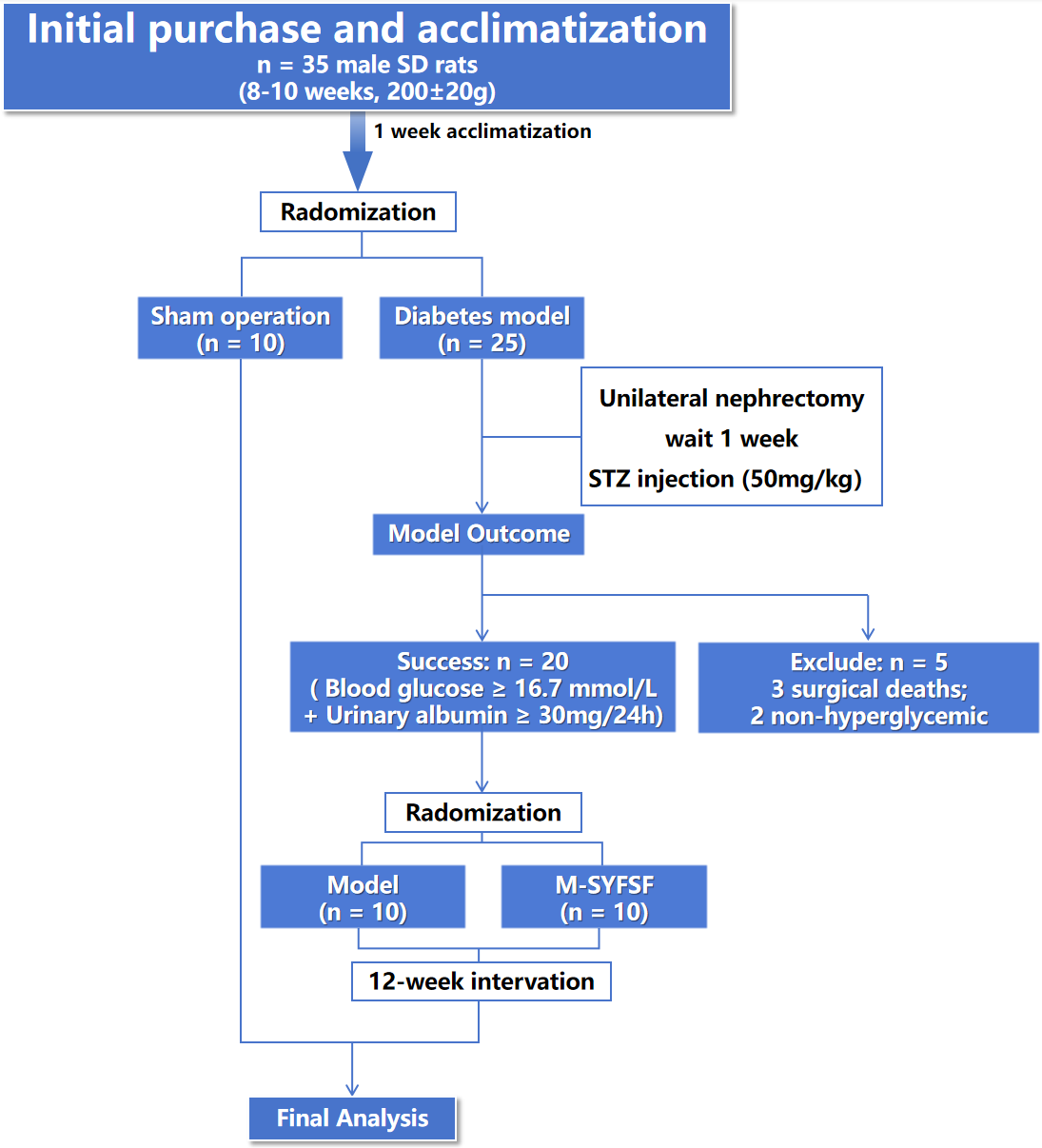
**

**Supplementary Figure S2. M-SYFSF reduces serum IL-6 levels in diabetic kidney disease.**

Serum IL-6 levels were measured by ELISA (Bioswamp, RA20607) at the end of 8-week treatment. Data are mean ± SEM (n = 6/group). ***p < 0.001 vs. Control; ###p < 0.001 vs. Model (one-way ANOVA with Tukey's test). M-SYFSF treatment (11.34 g/kg/day) significantly reduced serum IL-6 levels, demonstrating anti-inflammatory effects in the diabetic kidney disease model.


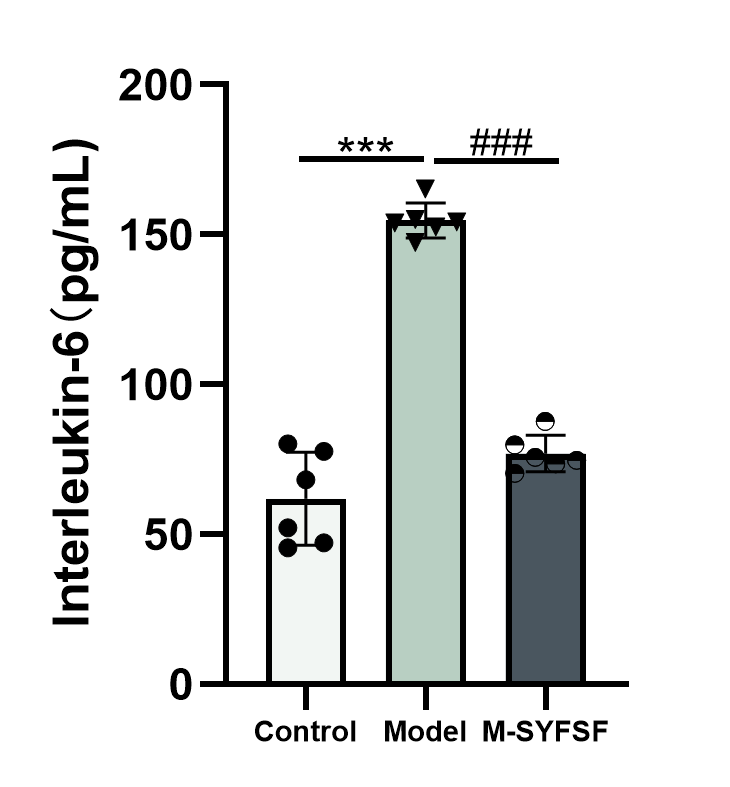

Supplement: Supplementary file 1 — Supplementary Material 1 [file 13020_2025_1298_MOESM1_ESM.docx]
